# Green synthesis of iron-doped cobalt sulfide *via* synergistic electronic and structural engineering in ethaline deep eutectic solvent for efficient oxygen evolution reaction

**DOI:** 10.1039/d5ra03467a

**Published:** 2025-09-01

**Authors:** Wenqiang Yang, Shaohua Wang, Wen Shi, Yakun Yin, Youpo Mise, Juan An, Xuejiao Zhou, Wentang Xia

**Affiliations:** a School of Metallurgy and Power Engineering, Chongqing University of Science and Technology Chongqing 401331 P. R. China wenqiangyang@cqust.edu.cn wentangx@163.com +86-023-65023711 +86-023-65023711; b Chongqing Municipal Key Laboratory of Institutions of Higher Education for Value-added Treatment and Green Extraction from Complicated Resources Chongqing 401331 P. R. China

## Abstract

The development of high-efficiency, earth-abundant electrocatalysts for the oxygen evolution reaction (OER) is essential for scalable green hydrogen production, yet challenges persist in balancing activity, stability, and cost. Herein, we present a sustainable approach to synthesize Fe-doped cobalt sulfide (Co–S–30Fe) nanoparticles using an ethaline deep eutectic solvent-mediated strategy, which enables precise control over Fe incorporation to optimize both structural and electronic properties. The engineered Co–S–30Fe/NF electrode exhibited exceptional OER performance in alkaline media, requiring an overpotential of only 278 mV at 100 mA cm^−2^, with a Tafel slope of 44.6 mV dec^−1^ and outstanding operational stability. Spectroscopic analyses revealed that Fe^3+^ doping induces three synergistic effects: (1) coexistence of dynamically active Co^2+^/Co^3+^ and Fe^2+^/Fe^3+^ redox couples, (2) substantial oxygen vacancy generation, and (3) ethaline-directed self-assembly of monodisperse nanospheres (∼96 nm) with 31.6% higher electrochemical surface area. This synergy of electronic reconstruction, defect engineering, and morphology control significantly enhances charge transfer kinetics (67% reduction in charge-transfer resistance) and intrinsic catalytic activity (4.4-fold increase in turnover frequency) compared to undoped Co–S. Critically, *in situ* electrochemical reorganization during the OER induced a surface transformation into oxygen-rich Co(Fe)–O/OH species, addressing the activity–stability trade-off. When integrated into a Co–S–30Fe/NF‖Pt/C/NF electrolyzer, the system achieved overall water splitting at low cell voltages of 1.53 V and 1.75 V (10 and 100 mA cm^−2^, respectively) while maintaining stable operation for 100 h at 10 mA cm^−2^.

## Abstract 2

The development of high-efficiency, earth-abundant electrocatalysts for the oxygen evolution reaction (OER) is essential for scalable green hydrogen production, yet challenges persist in balancing activity, stability, and cost.

## Graphical abstract

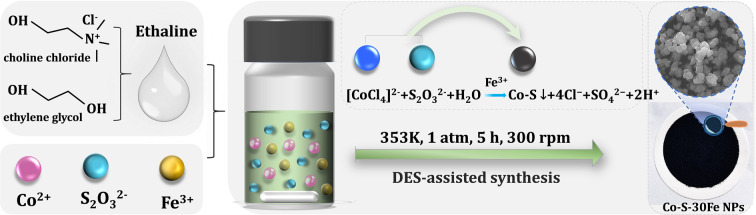

## 1. Introduction

Hydrogen energy stands as a pivotal enabler for achieving carbon neutrality. Its green, large-scale production hinges on breakthroughs in water electrolysis technology.^1–3^ However, the sluggish kinetics of the anodic oxygen evolution reaction (OER) severely constrain hydrogen generation efficiency, driving an urgent need for cost-effective, high-performance non-precious metal OER electrocatalysts.^4–7^ Deep Eutectic Solvents (DESs), comprising hydrogen-bonded complexes of acceptors (HBAs) and donors (HBDs), have emerged as innovative green media for energy material synthesis.^8^ Their tunable solvation environments, topological adaptability, intrinsic self-templating properties, low volatility, high thermal stability, biodegradability, and exceptional solvation power establish a versatile platform for sustainable electrocatalyst design.^9^ Within this platform, DESs concurrently function as solvents, templates, and reductants/catalysts, facilitating instantaneous nucleation, morphology-specific nanostructure control, heteroatom doping, and environmentally benign product isolation.^10^ Recent advances in DES-based OER catalyst optimization focus on three core strategies:

(1) Morphological engineering and structural control: leveraging DESs' dynamic hydrogen-bond networks and spatial confinement effects enables precise tailoring of nanostructure dimensionality (0D to 3D) and morphology (*e.g.*, nanosheets, porous architectures, dendrites). This significantly enhances specific surface area and mass/electron transfer efficiency.^11–17^ Representative DES-directed syntheses include ultrathin nanosheets (amorphous NiFe nitrides,^11^ NiMnCo oxide networks^12^), nanocrystals with specific facet exposure (NiCo_2_O_4_ octahedra^13^), bicontinuous nanoporous channels (high-entropy alloys^14^), metal-hydroxide heterointerfaces,^15^ and dendritic NiCu alloys.^16^

(2) Compositional optimization and synergistic effects: the tunable coordination environment within DESs provides a powerful tool for precise chemical design.^18–27^ Key approaches encompass: (i) multimetallic synergy: regulating metal precursor types and ratios in DESs yields synergistic multimetallic or high-entropy materials,^14,17^ optimizing electronic structure and redox properties. (ii) Heteroatom doping: DESs serve as media for direct doping agents or controlled-release dopant sources, enabling effective incorporation of non-metal elements (*e.g.*, S^21^ N^27^) to modulate active site electronic states and stability. (iii) Defect engineering: controlled calcination atmospheres^19^ or solvent engineering^22,23^ induce oxygen vacancies and other defects, accelerating reaction kinetics. Examples include activating lattice oxygen mechanisms,^22,26^ inducing lattice distortion (*e.g.*, in SNO–C/Co_8_FeS_8_),^24^ and optimizing metal valence distributions (*e.g.*, Co^2+^/Co^3+ 22^, Fe^3+^/Ni^3+^).^23^

(3) Solvent engineering and sustainable processing: DESs' low volatility, high thermal stability, strong dissolution capability, and tunable physicochemical properties confer distinct advantages: (i) green synthesis: enables surfactant-free dispersion of ultrafine particles under ambient pressure.^28^ (ii) Enhanced stability: improves catalyst corrosion resistance (*e.g.*, against Cl^−^)^28^ and strengthens metal–support interactions.^29^ (iii) Templating and self-assembly: directs formation of specialized architectures (*e.g.*, self-assembled hollow microspheres^30^). (iv) Resource circularity: enables direct conversion of waste materials (*e.g.*, spent batteries) into high-performance catalysts,^31^ underscoring inherent sustainability. (v) Precise environment control: the DES coordination environment critically dictates product structure formation;^32,33^ additives like water allow fine-tuning (*e.g.*, for 2D heterostructure growth).^22,23^

Despite significant progress, persistent challenges include inefficient interfacial charge-transfer kinetics and catalyst dissolution under operational conditions. This work presents a green coordination engineering strategy for synthesizing Fe-doped cobalt sulfide nanoparticles with high OER performance in ethaline/DES. The optimized Co–S–30Fe catalyst exhibited synergistic effects across multiple scales through the following three mechanisms: (1) electronic modulation *via* Co^2+^/Co^3+^ and Fe^2+^/Fe^3+^ mixed valence states, strengthening metal–sulfur covalency; (2) oxygen vacancy enrichment, enhancing OER kinetics; (3) DES-directed growth of monodisperse nanospheres (∼96 nm), yielding a 31.6% increase in electrochemical active surface area (ECSA). These synergies resulted in exceptional OER performance for Co–S–30Fe/NF in 1 M KOH, delivering an ultralow overpotential of 278 mV at 100 mA cm^−2^ (61 mV lower than pristine Co–S), a Tafel slope of 44.6 mV dec^−1^, and robust stability with only 9 mV degradation over 27 h at 10 mA cm^−2^. Mechanistic studies correlated the performance enhancements to accelerated charge transfer kinetics (67% reduction in charge-transfer resistance, *R*_ct_) and improved intrinsic activity (4.4-fold increase in turnover frequency, TOF) compared to undoped Co–S. Importantly, *in situ* electrochemical reorganization during the OER induced a surface transformation into oxygen-rich Co(Fe)–O/OH species. When integrated into a Co–S–30Fe/NF‖Pt/C/NF electrolyzer, the system achieved overall water splitting at low cell voltages of 1.53 V and 1.75 V (10 and 100 mA cm^−2^, respectively), while maintaining stable operation for 100 h at 10 mA cm^−2^.

## 2. Experimental section

### 2.1. Chemicals and reagents

All chemicals and solvents, including cobalt(ii) chloride hexahydrate (CoCl_2_·6H_2_O, ≥99%), iron(iii) chloride hexahydrate (FeCl_3_·6H_2_O, ≥99%), sodium thiosulfate pentahydrate (Na_2_S_2_O_3_·5H_2_O, ≥99.5%), potassium hydroxide (KOH, ≥95%), sodium dihydrogen phosphate dihydrate (NaH_2_PO_4_·2H_2_O, ≥99%), disodium hydrogen phosphate dihydrate (Na_2_HPO_4_·2H_2_O, ≥98%), and deionized water (resistivity ≥18.2 MΩ cm at 298 K), were procured from Shanghai Aladdin Biochemical Technology Co., Ltd and used without further purification. Choline chloride (C_5_H_14_ClNO, ChCl, ≥98%) and ethylene glycol ((CH_2_OH)_2_, EG, ≥99.5%) were obtained from Shanghai Sinopharm Chemical Reagent Co., Ltd Commercial platinum on carbon (Pt/C, 20 wt%), ruthenium(iv) oxide (RuO_2_, ≥99%), and Nafion solution (5 wt%) were supplied by Sigma-Aldrich (St. Louis, USA). A 2.0 M phosphate-buffered saline (PBS, pH 7.0) solution was prepared by dissolving 0.038 g of NaH_2_PO_4_ and 0.062 g of Na_2_HPO_4_ in 50 mL deionized water under ambient conditions (298 K). The deep eutectic solvent (DES), termed ethaline, was synthesized by mixing ethylene glycol (EG) and choline chloride (ChCl) at a 2 : 1 molar ratio, followed by heating (313 K) and vigorous stirring until a homogeneous transparent liquid formed.

### 2.2. Synthesis of Fe-doped cobalt sulfide nanoparticle catalysts

A series of iron-doped cobalt sulfide nanoparticle catalysts (denoted as Co–S–*x*Fe NPs, where “*x*” represents the FeCl_3_ molar concentration) were synthesized *via* a facile, additive-free ethaline-assisted liquid-phase method. In a typical procedure, 0.3 M CoCl_2_·6H_2_O, 0.2 M Na_2_S_2_O_3_·5H_2_O, and varying concentrations of *x* mM FeCl_3_·6H_2_O (*x* = 0, 10, 20, 30, 40, 50) were dissolved in 50 mL ethaline under continuous stirring at 353 K for 5 h. The resultant black precipitate was collected by vacuum filtration, washed three times each with deionized water and ethanol, and dried overnight in a vacuum oven at 313 K under a negative pressure of −0.1 MPa. The final products were labeled as Co–S (*x* = 0) and Co–S–*x*Fe (*x* = 10, 20, 30, 40, 50), where the numerical prefix indicates the molar concentration of FeCl_3_. Nickel foam (NF) substrates (0.5 cm × 0.5 cm) were pretreated by ultrasonication in ethanol (10 min) and 10 vol% HCl (10 min), followed by rinsing with deionized water and air-drying. To prepare working electrodes, a homogeneous catalyst ink was formed by ultrasonically blending 5 mg catalyst (RuO_2_, Pt/C, Co–S, or Co–S–*x*Fe), 30 μL 5 wt% Nafion solution, 470 μL ethanol, and 500 μL deionized water for 1 h. Subsequently, 50 μL of the suspension was drop-cast onto the pretreated NF surface and dried at 333 K under vacuum for 12 h, yielding a catalyst loading of ∼1.0 mg cm^−2^. After systematic optimization of reaction parameters (Fig. S1–S4), the optimal synthesis conditions were determined as follows: 0.3 M CoCl_2_·6H_2_O, 0.2 M Na_2_S_2_O_3_·5H_2_O, and 30 mM FeCl_3_·6H_2_O in ethaline, reacted at 353 K with 300 rpm stirring for 5 h.

### 2.3. Electrochemical measurements of the as-prepared catalysts

Electrochemical evaluations were performed using a Princeton PARSTAT 4000 potentiostat (AMETEK, USA) in a three-electrode system with argon-saturated 1.0 M KOH electrolyte (298 K, pH ∼13.9). The catalyst-loaded NF (0.5 cm × 0.5 cm), a carbon rod (*Φ* = 6 mm), and a Hg/HgO electrode (1.0 M KOH filled) served as the working, counter, and reference electrodes, respectively. Oxygen evolution reaction (OER) activities were assessed by linear sweep voltammetry (LSV) at a scan rate of 5 mV s^−1^. The electrode potential was converted to the reversible hydrogen electrode (RHE) scale using *E* (*vs.* RHE) = *E* (*vs.* Hg/HgO) + 0.0592 × pH + 0.098 V. Electrochemical impedance spectroscopy (EIS) was conducted in 1.0 M KOH solution over a frequency range of 100 kHz to 0.1 Hz with an AC amplitude of 5 mV, and data were fitted using a simplified Randles equivalent circuit. All polarization curves were *iR*-corrected (90% compensation) based on the solution resistance (*R*_s_) derived from EIS. Turnover frequency (TOF) of the catalysts was calculated as TOF = *I*/(4*Fn*), where *I* (A) is the current at a selected potential, *F* is the Faraday constant (96 485 C mol^−1^), and *n* (mol cm^−2^) represents the active site density. The *n* value was determined by integrating the charge (*Q*, C) from cyclic voltammetry (CV) scans in 2.0 M PBS using *n* = *Q*/(4*F*). Electrochemically active surface area (ECSA) was estimated *via* double-layer capacitance (*C*_dl_) measurements using CV scans in non-faradaic regions. Stability tests included CV cycling (100 mV s^−1^), multi-step chronoamperometry, and chronopotentiometry at fixed current densities.

### 2.4. Structural characterization

Fourier-transform infrared (FTIR) spectra of the ethaline/DES system were recorded on a Bruker IFS-66 spectrometer (Germany) in the 400–4000 cm^−1^ range with a resolution of 4 cm^−1^. Grazing-incidence X-ray diffraction (XRD) patterns were acquired using a Rigaku D/max-2500PC diffractometer (Cu-Kα radiation, *λ* = 0.15418 nm) with a 2*θ* range of 10–90°. Morphological and elemental analyses were performed *via* field-emission scanning electron microscopy (FESEM, JEOL JSM-7800F, 5 kV). X-ray spectroscopy (EDS) was employed to observe morphology and analyze elemental distribution. Transmission electron microscopy (TEM) and high-resolution TEM (HRTEM) images were obtained using a Tecnai G2 F30 microscope (FEI, Netherlands) at 200 kV. X-ray photoelectron spectroscopy (XPS) data were collected on a PHI 550 VersaProbe spectrometer (Al Kα source), with binding energies calibrated against the C 1s peak at 284.6 eV. Room-temperature photoluminescence (PL) spectroscopy measurements were obtained using a HITACHI F-4500 fluorescence spectrophotometer. Elemental composition analysis of the electrolyte was determined by inductively coupled plasma optical emission spectrometry (ICP-OES) using a PerkinElmer Optima 2000 DV spectrometer. Electron paramagnetic resonance (EPR) spectroscopy was recorded on a JES-FA300 spectrometer (JEOL) under low-temperature conditions (77 K), with a TE0112 cylindrical cavity and a magnetic field control range of 0 T to 2.0 T.

## 3. Results and discussion

**Fig. 1:**
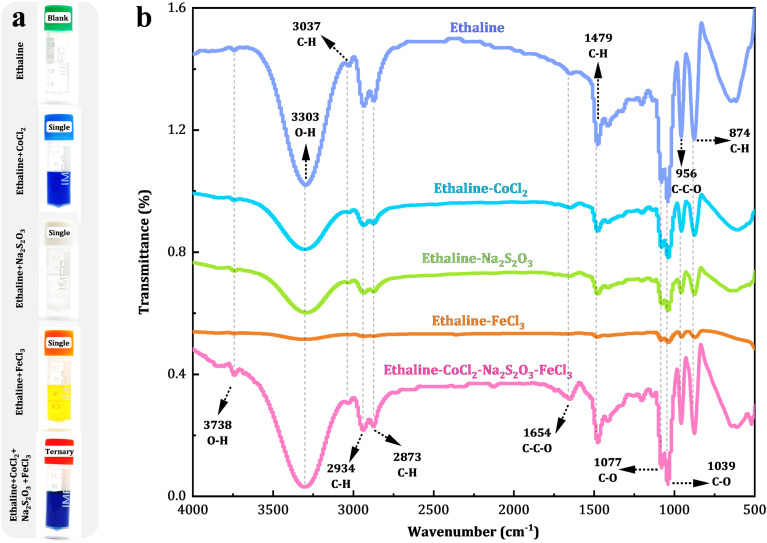
(a) Photographs and (b) FTIR spectra of blank ethaline, single-solute systems (0.30 M CoCl_2_ in ethaline, 0.20 M Na_2_S_2_O_3_ in ethaline, and 0.03 M CoCl_2_·6H_2_O in ethaline), and a ternary system (0.30 M CoCl_2_ + 0.02 M Na_2_S_2_O_3_ + 0.03 M CoCl_2_·6H_2_O in ethaline).

A deep eutectic solvent (DES) composed of ethylene glycol (EG) and choline chloride (ChCl) at a 2 : 1 molar ratio, known as ethaline, formed a homogeneous colorless liquid (Fig. 1a). Upon introducing 0.30 M CoCl_2_·6H_2_O, the solution transitioned to a vivid blue transparent state, consistent with the characteristic d–d electronic transitions of the tetrahedral [CoCl_4_]^2−^ complexes formed by Co^2+^ and Cl^−^ ions.^34^ Addition of 0.20 M Na_2_S_2_O_3_·5H_2_O induced a milky colloidal appearance, indicating the potential formation of colloidal structures due to microdomain aggregation of thiosulfate (S_2_O_3_^2−^), which caused the solution to appear turbid. The introduction of 30 mM FeCl_3_·6H_2_O yielded a bright yellow transparent solution, attributed to the formation of low-coordinate chlorinated iron complexe of [FeCl_4_]^−^.^35^ When CoCl_2_, Na_2_S_2_O_3_, and FeCl_3_ coexisted in ethaline, the solution exhibited a deep blue coloration, suggesting synergistic modifications in the electronic environments of metal centers through competitive coordination of Cl^−^ and S-containing ligands, accompanied by intermetallic or ligand-mediated electronic interactions.

FTIR spectroscopy (Fig. 1b) provided critical insights into the hydrogen-bonding network of the ethaline system. The broad absorption band spanning 3500–3000 cm^−1^ corresponds to O–H stretching vibrations within the ChCl-EG hydrogen-bonded framework.^36,37^ Notably, the broadened absorption peak at 3303 cm^−1^ exhibits a pronounced red shift (Δ*ν* ≈ 197 cm^−1^) compared to the free hydroxyl groups in pure EG (∼3500 cm^−1^). This shift is attributed to the strong hydrogen-bond acceptor role of Cl^−^, forming O–H⋯Cl^−^ interactions with the hydroxyl groups of EG. Additionally, some hydroxyl groups of ChCl may participate in O–H⋯O hydrogen bonds, collectively constructing a dense hydrogen-bond network.^38^ The weak peak at 3037 cm^−1^ is assigned to the C–H stretching vibrations of the methylene (CH_2_) groups in ChCl, which exhibit a blue shift due to the electron-withdrawing effect of the quaternary ammonium cation, resulting in a higher vibrational frequency compared to typical aliphatic C–H vibrations (∼2925 cm^−1^).^39^ The faint peak at 3738 cm^−1^ likely indicates trace free hydroxyl groups or adsorbed moisture.

These findings indicate that ChCl and EG form a stable deep eutectic network through multiple hydrogen bonds, contributing to its low melting point and high ionic conductivity. Furthermore, characteristic peaks at 2936 and 2871 cm^−1^ correspond to the C–H stretching vibrations of methylene groups in EG, while the peak at 882 cm^−1^ corresponds to the C–C skeletal vibrations in EG;^40,41^ The characteristic peaks of ChCl include the C–H in-plane bending vibration at 1480 cm^−1^, the asymmetric C–O stretching vibrations at 1036 and 1081 cm^−1^, the C–C–O coupling vibrations at 1640 and 953 cm^−1^, and the C–H stretching vibration at 862 cm^−1^.^42–45^ Crucially, the introduction of Co^2+^, S_2_O_3_^2−^, or Fe^3+^ induced neither additional peaks nor significant shifts in the FTIR profile of ethaline (Fig. S5), confirming the robust hydrogen-bonding stability of the DES matrix. While these ions may locally perturb the network through coordination or electrostatic effects, the dynamic hydrogen-bonding reorganization maintains vibrational mode integrity. This inherent ionic tolerance enables confined nanoparticle nucleation and growth, positioning DESs as ideal templates for size-controlled synthesis.^46,47^

Exploiting ethaline's dynamic yet stable hydrogen-bonding microenvironment, we systematically optimized synthetic parameters (FeCl_3_·6H_2_O concentration, bath temperature, reaction duration, and stirring speed) to engineer Fe-doped cobalt sulfide nanomaterials with superior OER activity. The optimal performance was achieved at 30 mM FeCl_3_, 353 K, 5 h, and 300 rpm (Fig. S1–S4). The FeCl_3_ concentration directly influences the nucleation kinetics and growth pathways of the active intermediates. At 30 mM FeCl_3_ (Fig. S1), the coordination competition effect optimizes the interfacial exchange rate between [CoCl_4_]^2−^ and S^2−^, enabling precise control over product morphology and structure. Ethaline's inherent high viscosity necessitated the synergistic regulation of thermodynamic and mass transfer processes, where 353 K balanced ionic diffusion enhancement (Fig. S2) with nucleation rate suppression, ensuring monodisperse nanoparticle formation. Reaction duration critically influenced phase evolution (Fig. S3), with incomplete thiosulfate (S_2_O_3_^2−^) disproportionation dominating at short reaction times (<5 h), whereas prolonged durations (>5 h) caused Ostwald ripening and sulfur over-incorporation, both of which compromised active-site density and metal–sulfur covalency. Furthermore, controlled laminar mixing at 300 rpm homogenized reaction microenvironments through shear-stress modulation (Fig. S4), circumventing turbulent local gradients that drive heterogeneous nucleation.

**Fig. 2:**
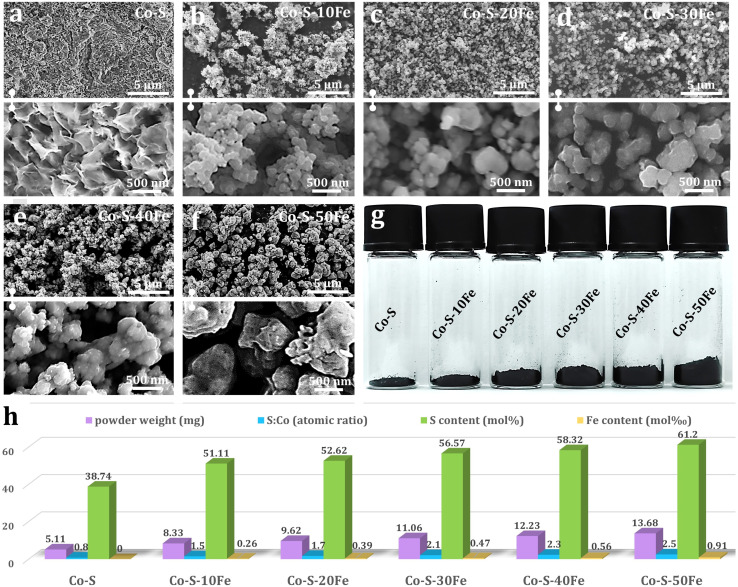
(a–f) FESEM images of Co–S (*x* = 0 mM) and Fe-doped Co–S (Co–S–*x*Fe, *x* = 10–50 mM) samples synthesized from a 0.30 M CoCl_2_–0.20 M Na_2_S_2_O_3_–*x* mM FeCl_3_·6H_2_O–ethaline system. (g) Photographs of the Co–S and Co–S–*x*Fe samples. (h) Quantitative analysis of powder mass (gravimetric measurement), S/Co molar ratio, sulfur content, and iron content, as determined by ICP-OES.

To elucidate the regulatory mechanisms of FeCl_3_ concentration on the morphology and composition of Co/Fe–S materials, a systematic investigation was conducted using a gradient doping strategy. A ternary reaction system comprising 0.30 M CoCl_2_–0.20 M Na_2_S_2_O_3_–*x* mM FeCl_3_·6H_2_O–ethaline (*x* = 0, 10, 20, 30, 40, 50) was established under a fixed synthesis condition (353 K, 300 rpm, 5 h). The resultant materials, denoted as Co–S (*x* = 0) and Co–S–*x*Fe (*x* = 10–50 mM), exhibited distinct morphological evolution governed by Fe^3+^-mediated kinetic control (Fig. 2a–f). In the absence of FeCl_3_ (Fig. 2a), the Co–S sample displayed irregular nanosheets (∼15 nm thick) that formed densely packed aggregates. This morphology can be attributed to rapid nucleation and anisotropic growth driven by direct ligand exchange between [CoCl_4_]^2−^ and S_2_O_3_^2−^. Upon introducing 10 mM FeCl_3_ (Fig. 2b), a notable morphological transition occurred, resulting in loosely aggregated *quasi*-spherical nanoparticles (∼78 nm), indicative of Fe-mediated surface reconstruction. Further increasing FeCl_3_ to 20–30 mM (Fig. 2c and d) yielded monodisperse spherical nanoparticles (92–128 nm) with enhanced size uniformity, signifying an optimal kinetic balance in nucleation-growth dynamics. The substantial improvement in particle size uniformity confirms the synergistic regulatory effect of Fe^3+^ and the ethaline solvent. This morphological transition is driven by synergistic mechanisms: (1) Fe^3+^ forms a stable [FeCl_4_]^−^ tetrahedral complex with Cl^−^, which competitively weakens the Co–Cl bonds and accelerates S^2−^ substitution, thereby increasing nucleation density while suppressing oriented crystal growth; (2) Fe^3+^ likely adsorbs electrostatically on the nascent crystal surfaces, creating electrostatic barriers. Additionally, ethaline's hydrogen-bond-mediated steric hindrance effectively inhibits Ostwald ripening. Beyond 30 mM (40–50 mM, Fig. 2e and f), particle aggregation re-emerged with increased average diameters (∼157 nm at 40 mM and ∼214 nm at 50 mM), suggesting destabilization of the colloidal system at elevated Fe^3+^ concentrations.

Interestingly, the mass of the black products increased with the addition of FeCl_3_ (Fig. 2g). Fig. 2h summarizes the corresponding data regarding powder mass, S/Co atomic ratio, sulfur molar content, and iron molar content. Monitoring revealed a nonlinear increase in product yield (5.1 → 13.8 mg) with increasing FeCl_3_ concentration (0 → 50 mM), demonstrating a 2.7-fold increase in product mass but a decreasing growth rate (62.7% → 9.5%). At low Fe^3+^ concentrations (≤30 mM), the Lewis-acidic Fe^3+^ facilitated the protonation and decomposition of S_2_O_3_^2−^, leading to enhanced generation of active S^2−^ species and a more optimized distribution of nucleation sites, which ultimately improved precursor utilization efficiency. Conversely, above 30 mM, ethaline's high viscosity (16.8 cP at 353 K)^48^ restricts ionic diffusion, while Fe^3+^-Cl^−^ complexation reduces free ligand availability, causing some Co^2+^ to remain in solution as octahedral [Co(H_2_O)_6_]^2+^,^49^ thereby decreasing the proportion of metal ions participating in the sulfide reaction. ICP-OES analysis revealed the S/Co atomic ratio increased progressively from 0.8 (0 mM FeCl_3_) to 2.5 (50 mM FeCl_3_) with Fe doping, driven by dual mechanisms: (1) Fe^3+^-enhanced sulfur precursor decomposition elevates reactive sulfur species; (2) local structural perturbations induced by Fe^3+^ incorporation facilitate sulfur accommodation at interstitial sites. Notably, although the Fe/Co molar ratio in the precursor reached 16.7% (50 mM condition), product Fe content remains low (0.91 at%), attributed to [FeCl_4_]^−^ complex stabilization in ethaline's chloride-rich environment, which suppresses Fe incorporation into the solid phase.^50^ These findings establish that Fe^3+^ concentration critically governs nucleation-growth pathways through competitive coordination and interfacial effects. Below 30 mM, Fe^3+^ promotes the formation of monodisperse Co–S spherical nanoparticles *via* optimized kinetic control, while higher concentrations induce particle coarsening through diffusion limitations and bridging aggregation. This gradient doping strategy demonstrates precise morphological and compositional tuning of transition metal sulfides in deep eutectic solvent systems.

**Fig. 3:**
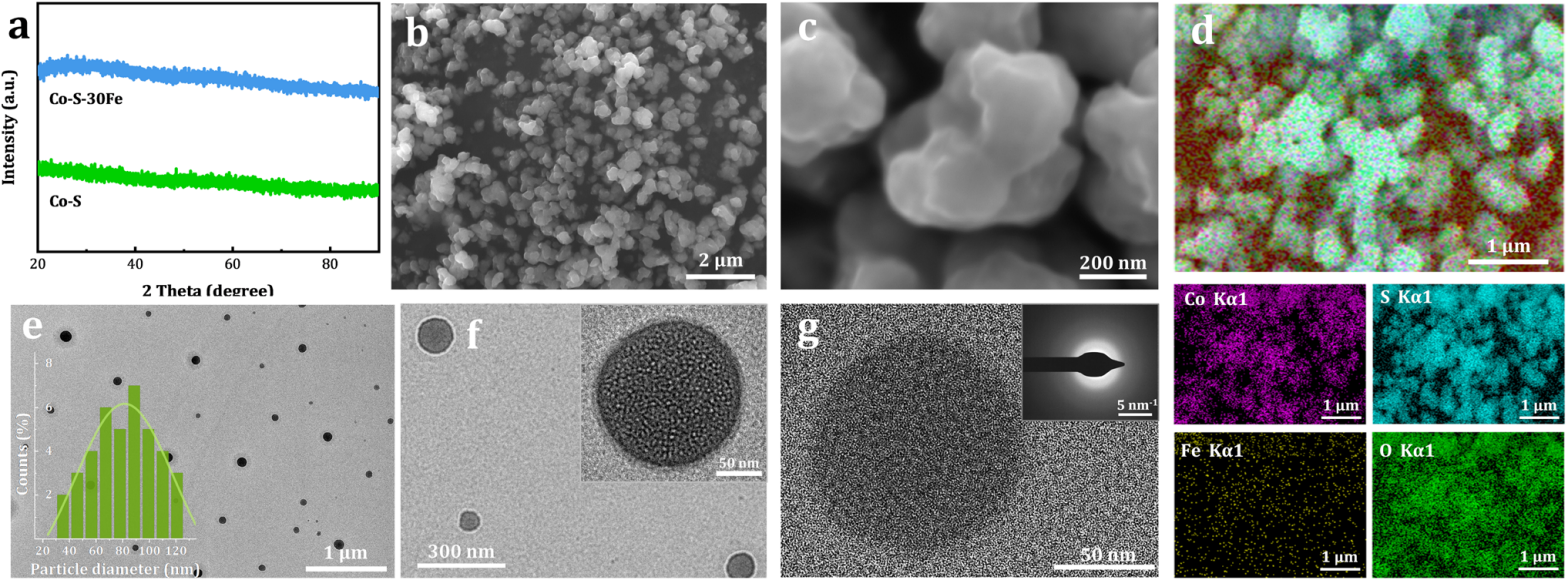
(a) XRD patterns of Co–S and Co–S–30Fe samples. (b and c) FESEM images of Co–S–30Fe. (d) EDS elemental mapping of Co–S–30Fe. (e) TEM images of Co–S–30Fe NPs (inset: particle size distribution). (f) Magnified TEM image of Co–S–30Fe NPs. (g) HRTEM image of Co–S–30Fe NPs (inset: SAED pattern).

The structural evolution of the Fe-free Co–S and Fe-doped Co–S–30Fe materials is depicted in Fig. 3a. Within the 2*θ* range of 20° to 90°, the XRD pattern of the undoped Co–S sample shows no discernible diffraction peaks, indicating its amorphous structure. Notably, Fe doping did not induce crystallization of the Co–S species. FESEM analysis (Fig. 3b and c) reveals that Co–S–30Fe consists of uniformly distributed nanoparticles with consistent particle size. Quantitative ICP-OES analysis shows a S/Co atomic ratio of 2.1 (Table S1), with an Fe content of 0.91 at%. Elemental mapping (Fig. 3e and f) reveals homogeneous distribution of Co, S, Fe, and O across the nanospheres, confirming uncompromised elemental homogeneity despite Fe incorporation and directly validating the successful synthesis of Fe-doped Co–S compounds. TEM characterization (Fig. 3e–g) confirms the spherical morphology and amorphous nature of the Co–S–30Fe, with an average particle size of ∼96 nm. The HRTEM images show an absence of lattice fringes, and the selected-area electron diffraction pattern (inset in Fig. 3g) exhibits only diffuse rings, consistent with the XRD results.

**Fig. 4:**
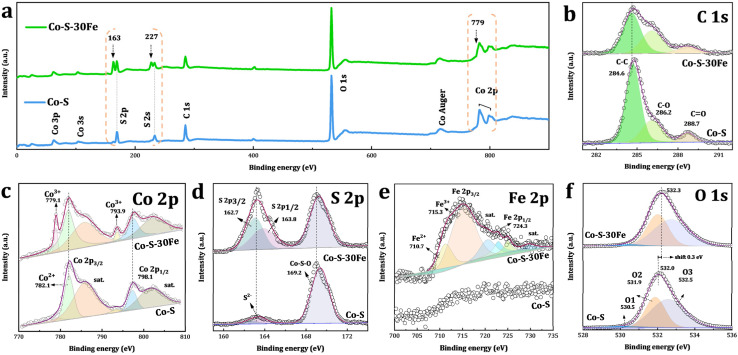
High-resolution XPS spectra of Co–S and Co–S–30Fe samples: (a) survey scan, (b) C 1s, (c) Co 2p, (d) S 2p, (e) Fe 2p, and (f) O 1s regions.

To further explore the modulation effects of Fe doping on the material's electronic structure, X-ray photoelectron spectroscopy (XPS) was employed to compare and analyze the surface chemical state evolution of Co–S and Co–S–30Fe materials. The survey spectra (Fig. 4a) reveal characteristic peaks of Co, S, O, and C elements in both samples. The C 1s signal primarily originates from the surface-adsorbed carbonaceous species. Notably, the Fe 2p signal intensity in Co–S–30Fe remains below detection limits, consistent with ICP-measured low Fe content (0.47 at%, Fig. 2h), suggesting Fe^3+^ is likely incorporated into the Co–S matrix through interstitial doping rather than surface physical adsorption. The C 1s high-resolution spectrum (Fig. 4b) exhibits three characteristic peaks at 284.6, 286.2, and 288.7 eV, corresponding to sp^2^ hybridized carbon (C–C/C=C), hydroxyl/ether groups (C–O), and carbonyl (C=O) functional groups, respectively. The relative intensity ratio of C–O/C=O (3.5) is significantly higher than that of graphite-based carbon materials (typically <0.1), indicating that the hydroxyl network in the ethaline solvent forms an organic–inorganic hybrid interfacial layer on the nanoparticle surface through hydrogen bonding. This interfacial engineering suppresses particle aggregation through steric hindrance while simultaneously provides abundant oxygen-containing functional groups (*e.g.*, C–O) that facilitate proton transport to enhance mass transfer kinetics during catalytic reactions.

Fe doping-induced electronic restructuring is seen in the Co 2p spectrum (Fig. 4c). Undoped Co–S shows characteristic Co^2+^ signatures at 782.1 eV (2p_3/2_) and 798.1 eV (2p_1/2_), with strong satellite peaks at 785.9 eV (Δsat(2p_3/2_) = 3.8 eV) and 802.9 eV (Δsat(2p_1/2_) = 4.8 eV). The reduced satellite splitting compared to CoO (Δsat ≈ 5–6 eV) indicates stronger Co–S covalency, matching the high-spin d^7^ configuration in Co–S phases.^51,52^ Fe incorporation introduces new peaks at 779.1 eV and 793.9 eV, attributed to Co^3+^ species formation through charge transfer-driven electronic reconstruction. This coexisting Co^2+^/Co^3+^ dual-valent system enhances metal–sulfur bond covalency while establishing an optimized electronic structure for improved charge transfer and active site distribution, which is crucial for improving intrinsic electrocatalytic activity of the material. The observed valence transition originates from Fe^3+^ → Co^2+^ electron transfer driven by the higher electronegativity of Fe^3+^. Quantitative analysis of the S 2p spectrum (Fig. 4d) further supports this mechanism, showing 6.4-fold enhancement in metal-sulfide peak intensity (162.7 eV S 2p_3/2_, 163.8 eV S 2p_1/2_) for Co–S–30Fe compared to Co–S. The elevated S/Co ratio (2.9, Fig. 2h) confirms Fe^3+^-catalyzed S_2_O_3_^2−^ disproportionation to active S^2−^, strengthening Co–S bond covalency. Additionally, the characteristic 1.1 eV splitting between 162.7 eV (S 2p_3/2_) and 163.8 eV (S 2p_1/2_) confirms predominant Co–S speciation.^53,54^ The peak at 168.5 eV suggests the presence of SO*x* species, likely arising from partial oxidation of surface sulfur atoms. The high-resolution Fe 2p spectrum of Co–S–30Fe (Fig. 4e) provides clear evidence for the coexistence of Fe^2+^ and Fe^3+^ oxidation states. The Fe^2+^ species are characterized by spin–orbit doublets at 710.7 eV (Fe 2p_3/2_) and 722.9 eV (Fe 2p_1/2_), while the Fe^3+^ species exhibit corresponding peaks at 715.3 eV and 725.0 eV.^55^ The formation of this mixed valence state system can be attributed to partial Fe^3+^ reduction by EG's reductive hydroxyl groups and thiosulfate, as well as the electronic transfer from Co^2+^ to Fe^3+^. This mixed-valent system facilitates enhanced charge transfer kinetics through Fe^2+^/Fe^3+^ redox couples, improving electrical conductivity and catalytic activity. The O 1s spectrum (Fig. 4f) of Co–S shows three oxygen-related peaks, 530.5 eV (O1) corresponding to lattice oxygen (O^2−^), 531.9 eV (O2) attributed to surface hydroxyl groups (–OH) or oxygen vacancies, and 532.5 eV (O3) arising from adsorbed water or organic residues.^56^ Quantitative analysis of peak fitting reveals that after Fe doping (Co–S–30Fe), the relative intensity of the O2 component increases slightly from 36.7% to 39.7% (*Δ* = 3.0%), with a 0.3 eV shift in binding energy, suggesting that Fe doping induces more oxygen vacancy generation. To verify oxygen vacancy formation, low-temperature (77 K) EPR spectroscopy of Co–S–30Fe (Fig. S6) revealed a prominent symmetric signal at *g* = 2.002, characteristic of unpaired electrons at oxygen vacancy sites.^57–60^ Critically, comparative PL spectra (Fig. S7) demonstrated markedly enhanced intensities at 404.3 nm (assigned to V_O_˙) and 468.0 nm (V_O_˙) for Co–S–30Fe *versus* undoped Co–S.^61,62^ This intensity increase confirms a substantially higher oxygen vacancy concentration induced by Fe doping.

**Scheme 1:**
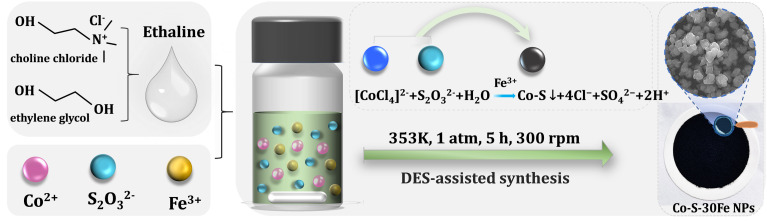
Ethaline-assisted one-step synthesis of Fe-doped Co–S (Co–S–30Fe) in a 0.20 M CoCl_2_–0.15 M Na_2_S_2_O_3_–ethaline system at 353 K, 1 atm, for 5 h with stirring at 300 rpm.

Integrated analysis through FTIR coordination studies, FESEM/TEM morphology characterization, EDS elemental mapping, and XPS bonding features reveals a synergistic “coordination equilibrium–sulfur activation–interface confinement” mechanism governing the formation of Co–S–30Fe nanoparticles in ethaline (Scheme 1). In the 0.3 M CoCl_2_·6H_2_O–0.2 M Na_2_S_2_O_3_·5H_2_O–30 mM FeCl_3_·6H_2_O–ethaline system, Co^2+^ ions preferentially coordinate with Cl^−^ to form thermodynamically stable [CoCl_4_]^2−^ tetrahedral complexes.^63,64^ Simultaneously, S_2_O_3_^2−^ anions adsorb onto the [CoCl_4_]^2−^ surface *via* hydrogen bonding and electrostatic interactions with choline (Ch^+^) cations, creating oriented precursor assemblies.^65,66^ Significantly, the appropriate amount of Fe^3+^ plays a crucial role in the controlled synthesis of Co–S–30Fe nanoparticles. It is proposed that Fe^3+^ modulates the reaction pathway through three synergistic mechanisms: (i) Fe^3+^ competes with Co^2+^ for Cl^−^ ligands, destabilizing the thermodynamically stable [CoCl_4_]^2−^ complexes (eqn (1)). This competition enhances the release of free Co^2+^ ions, creating a localized metal ion concentration gradient that promotes nucleation. (ii) Trace amounts of free Fe^3+^ ions electrostatically adsorb onto the S_2_O_3_^2−^ surfaces, weakening the S–O bond and catalyzing its disproportionation into S^2−^ and SO_4_^2−^ (eqn (2)). (iii) Partial reduction of Fe^3+^ to Fe^2+^ by ethaline's EG and S_2_O_3_^2−^ forms self-sustaining Fe^2+^/Fe^3+^ redox pairs, driving efficient Co–S nucleation (eqn (3)) while maintaining a kinetically balanced reaction environment.

1 [CoCl_4_]^2−^ + Fe^3+^ → [FeCl_4_]^−^ + Co^2+^

2
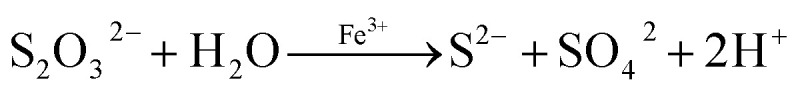

3 Co^2+^ + S^2−^ → Co − S↓

In the above process, the ionic radii of Fe^2+^/Fe^3+^ closely match that of Co^2+^, allowing Fe^2+^/Fe^3+^ to either substitute for Co^2+^ through topological adaptation or be doped interstitially into the Co–S phase, inducing local charge imbalance and leading to an increase in defect sites. This mixed-valent system enhances charge transfer efficiency through Fe^2+^/Fe^3+^ redox mediation while increasing active site density *via* Lewis acid-activated sulfur species. Concurrently, oxygen vacancies formed during doping serve as proton adsorption sites, optimizing oxygen evolution kinetics. Noticeably, the ethaline medium enables orchestrates nanoparticle growth through a triple-regulation mechanism: (i) dynamic hydrogen-bond networks impose steric hindrance against particle aggregation; (ii) high viscosity decelerates ion diffusion, modulating nucleation-growth kinetics; and (iii) competitive coordination between Cl^−^ and S^2−^ directs short-range structural ordering, tailoring the amorphous matrix's nanoscale architecture. This hierarchical “coordination-dissociation-reconstruction” reaction pathway, in synergy with the confinement effects of DES, results in well-dispersed Fe-doped Co–S nanomaterials with optimized morphology and electronic structures for enhanced electrocatalytic performance.

**Fig. 5:**
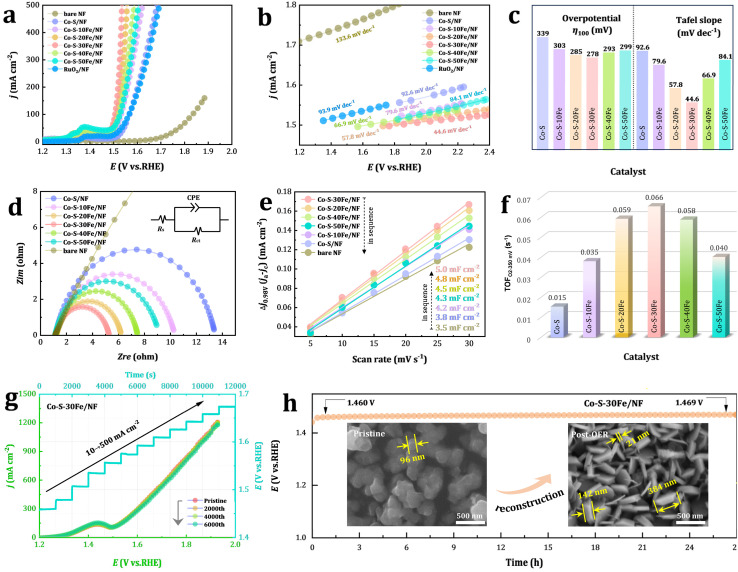
(a) Polarization curves for Co–S/NF, Co–S–*x*Fe/NF, bare NF substrate, and RuO_2_/NF after *iR* correction at a scan rate of 5 mV s^−1^. (b) Corresponding Tafel slopes for the above materials. (c) Comparison of overpotentials at 100 mA cm^−2^ and Tafel slopes for Co–S/NF and Co–S–*x*Fe/NF. (d) EIS Nyquist plots recorded at an overpotential of 350 mV (*vs.* RHE) for Co–S/NF and Co–S–*x*Fe/NF. (e) The differences in current density at 0.98 V *vs.* RHE (Δ*j* = *j*_a_ − *j*_c_) plotted against scan rate. (f) TOF calculated from the overpotential at *η*_OER_ = 350 mV for Co–S/NF and Co–S–*x*Fe/NF. (g) Multi-step chronopotentiometric curves for Co–S–30Fe/NF, tested sequentially at 10 and 20 mA cm^−2^ (each for 1000 s), followed by incremental steps from 50 to 500 mA cm^−2^ in 50 mA cm^−2^ intervals (each for 1000 s), and LSV curves of Co–S–30Fe/NF before and after 2000, 4000, and 6000 CV cycles (without *iR* correction). (h) Long-term chronopotentiometric stability test for Co–S–30Fe/NF at 10 mA cm^−2^ over 27 h. The inset SEM images show the structure changes of post-OER Co–S–30Fe/NF after 27 h OER electrolysis at *j* = 10 mA cm^−2^ in 1.0 M KOH at 298 K.

Building upon the clarified structural characteristics of the material, the OER performance of FeCl_3_·6H_2_O concentration gradients on Co–S–*x*Fe/NF electrodes was systematically investigated, focusing on Fe-induced electronic structure reconstruction and interfacial engineering synergies. Linear sweep voltammetry (LSV) in 1.0 M KOH at a scan rate of 5 mV s^−1^ revealed that Co–S–30Fe/NF exhibits superior OER activity, requiring an overpotential of only 278 mV at 100 mA cm^−2^ (Fig. 5a). This represents a 61 mV improvement over the undoped Co–S/NF (339 mV) and even surpasses the benchmark RuO_2_/NF (330 mV, Δ*η* = 52 mV). Tafel slope analysis further confirms the optimized reaction kinetics induced by Fe doping (Fig. 5b). Co–S–30Fe/NF exhibited the lowest Tafel slope (44.6 mV dec^−1^), 48.2% lower than that of Co–S/NF (92.6 mV dec^−1^), indicating a reduced activation energy barrier for the OER. These combined metrics (low *η*_10_ and small Tafel slope) for Co–S–30Fe/NF exceed not only commercial RuO_2_/NF but also most recently reported OER catalysts synthesized in DESs and operating in alkaline media (Fig. 2h and Table S2). Further analysis of the concentration-dependent overpotentials and Tafel slopes (Fig. 5c) reveals a distinct “volcanic” trend in the activity enhancement. The overpotential decreased monotonically from 339 mV to 278 mV as Fe^3+^ loading increased from 0 to 30 mM, followed by performance degradation beyond this threshold. Accordingly, this trend correlates with changes in material morphology (Fig. 2) and the S/Co atomic ratio (Fig. 2h), highlighting the critical role of Fe doping in synergistically tuning electronic structures and active site configurations. Electrochemical impedance spectroscopy (EIS) analysis (Fig. 5d) confirmed that Co–S–30Fe/NF possesses the lowest charge transfer resistance (*R*_ct_ = 4.14 Ω, Table S3), achieving a 3-fold enhancement in interfacial charge transfer efficiency compared to Co–S/NF (*R*_ct_ = 12.29 Ω). This improvement arises from: (1) enhanced intrinsic conductivity through Fe^3+^-induced Co^3+^/Co^2+^ and Fe^3+^/Fe ^2+^mixed-valence states (Fig. 4c and e), (2) optimized spherical morphology (Fig. 2d) shortening charge transport pathways; and (3) oxygen vacancy-enriched surfaces (Fig. 4f) facilitating hydroxyl ion adsorption/desorption.

Electrochemical active surface area (ECSA) and turnover frequency (TOF) analyses provided critical insights into intrinsic activity enhancement (Fig. 5e and f). The ECSA of the synthesized samples was estimated *via* simple CV method (details in Fig. S8) by measuring the electrochemical double layer capacitance, which is proportional to the ECSA. The ECSA of Co–S–30Fe/NF is 31.6% higher than that of Co–S/NF, consistent with the transition from agglomerated nanosheets to monodisperse nanospheres (Fig. 2d). Moreover, the OER TOF at an overpotential of 350 mV was quantified using the operando-determined active site density from CV charge integration (Fig. S9) to investigate the real catalytic active sites. The TOF of Co–S–30Fe/NF at 350 mV overpotential (0.066 s^−1^) surpassed that of Co–S/NF (0.015 s^−1^) by 4.4-fold, demonstrating a dual “quality-quantity” enhancement of active sites. To evaluate the OER durability of the Co–S–30Fe/NF electrode, long-term cyclic voltammetry and multi-step chronopotentiometric tests were performed. As shown in Fig. 5g, after sustained 2000, 4000, and 6000 CV cycles, the current density of Co–S–30Fe/NF remained virtually unchanged, and the potential response during multi-current switching was stable, indicating excellent OER stability. Furthermore, a 27 h stability test at 10 mA cm^−2^ showed a minimal potential increase of 9 mV (1.460 → 1.469 mV, Fig. 5h), underscoring robust structural integrity, high conductivity, and rapid mass transport properties. Notably, post-OER surface elemental analysis of Co–S–30Fe/NF (Fig. S10 and S11) revealed near-total sulfur depletion (55.37 → 0.86 at%, 98.45% loss) alongside significant oxygen accumulation (24.47 → 65.99 at%, +169.68%), with concurrent increases in cobalt (19.81 → 31.71 at%) and iron (0.35 → 1.44 at%) content. This elemental redistribution drives two synergistic reconstruction mechanisms: electrochemical sulfur oxidation triggers the phase transition from Co(Fe)–S to Co(Fe)–O/OH, while oxygen evolution-derived microbubbles generate interfacial shear forces that template nanosheet alignment and mesopore formation.^67^ Critically, XPS analysis (Fig. S12) confirms complete surface conversion to catalytically active Co^3+^ species (780.5 eV) with retention of residual S–Co coordination (161.5 eV), which stabilizes the metastable CoOOH/Co–S interface and enables exceptional electrochemical stability. The self-optimized Co–O/OH architecture integrates undercoordinated active sites with hierarchically porous nanosheet arrays, which collectively resolve the activity–stability trade-off through synergistic coordination of catalytic turnover kinetics and bubble-enhanced mass transport.

**Fig. 6:**
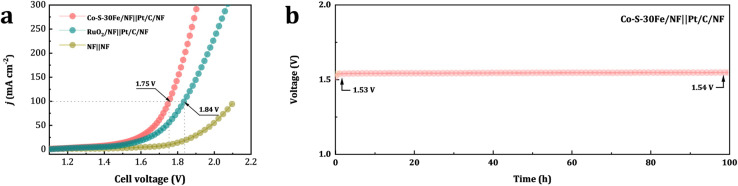
(a) LSV curves for overall water splitting using coupled Co–S–30Fe/NF‖Pt/C/NF, bare NF‖NF, and RuO_2_/NF‖Pt/C/NF at a scan rate of 5 mV s^−1^. (b) Long-term chronopotentiometric stability test of Co–S–30Fe/NF‖Pt/C/NF for overall water splitting at 10 mA cm^−2^ over 100 h.

Leveraging the exceptional OER activity and durability of Co–S–30Fe/NF, a dual-electrode electrolyzer (Co–S–30Fe/NF‖Pt/C/NF) was constructed for overall water splitting, using Co–S–30Fe/NF as the anode and Pt/C/NF as the cathode (Fig. 6a). The system achieved current densities of 100 mA cm^−2^ at a remarkably low cell voltage of 1.75 V, surpassing the noble-metal benchmark RuO_2_/NF‖Pt/C/NF (1.84 V). Furthermore, long-term constant-current chronopotentiometric tests for overall water splitting were conducted (Fig. 6b). The Co–S–30Fe/NF‖Pt/C/NF system reached 10 mA cm^−2^ at only 1.53 V, with a decay rate of just 0.7%, maintaining stable performance over more than 100 h.

The superior OER performance of Co–S–30Fe arises from a multiscale synergy enabled by Fe doping and sulfur incorporation: (i) Fe doping effectively tunes the electronic structure of Co–S, inducing the formation of Co^2+^/Co^3+^ and Fe^2+^/Fe^3+^ mixed-valence states. This electronic restructuring optimizes the OER reaction activity and kinetics rate; (ii) the introduction of Fe significantly increases the concentration of defect sites, which serve as active centers for water splitting and facilitate the proton-coupled electron transfer process, a critical aspect of OER kinetics; and (iii) Fe doping promotes the formation of monodispersed nanospheres, optimizing the morphology to enhance electrolyte penetration and shorten the charge and mass transport pathways, leading to increased electrochemical active surface area and turnover frequency. This hierarchical integration of electronic reconstruction, defect engineering, and morphological control enables Co–S–30Fe to outperform noble-metal benchmarks in both activity and stability.

## 4. Conclusions

This study presents an innovative strategy for synthesizing high-performance Fe-doped cobalt sulfide nanoparticles *via* a green ethaline-mediated strategy. By optimizing Fe^3+^ doping, the Co–S–30Fe catalyst achieved multiscale synergistic enhancements through three interconnected mechanisms: (1) electronic modulation *via* Co^2+^/Co^3+^ and Fe^2+^/Fe^3+^ mixed valence states, which strengthens metal–sulfur covalency to boost intrinsic OER activity; (2) oxygen vacancy enrichment, optimizing electron transfer pathways and increasing catalytically active site density; (3) DES-confined growth of monodisperse nanospheres (∼96 nm), yielding a 31.6% increase in ECSA. These synergies endowed the Co–S–30Fe/NF electrode with exceptional OER performance in 1 M KOH, exhibiting an ultralow overpotential of 278 mV at 100 mA cm^−2^, a Tafel slope of 44.6 mV dec^−1^, and remarkable stability with only 9 mV degradation over 27 h at 10 mA cm^−2^. Mechanistic analysis attributed these improvements to accelerated charge transfer kinetics (67% reduction in *R*_ct_) and enhanced intrinsic activity (4.4-fold increase in TOF) compared to undoped Co–S/NF. Notably, *in situ* electrochemical reorganization formed active oxygen-rich Co(Fe)–O/OH species on the surface, mitigating the activity–stability trade-off. When integrated into a Co–S–30Fe/NF‖Pt/C/NF electrolyzer, the system achieved overall water splitting at low cell voltages of 1.53 V and 1.75 V (10 and 100 mA cm^−2^, respectively) while maintaining stable operation for 100 h at 10 mA cm^−2^. This work not only provides a novel approach to designing high-efficiency non-precious OER catalysts but also highlights the unique advantages of DES-mediated synthesis in achieving precise control over nanomaterial morphology and electronic structure.

## Author contributions

Wenqiang Yang, Shaohua Wang, Wen Shi, Youpo Mise and Yakun Yin carried out experiments, Wenqiang Yang conceived the project, Wenqiang Yang, Wentang Xia, Xuejiao Zhou and Juan An supervised electric cell and electrochemical aspects of the project, and all authors cowrote the manuscript.

## Conflicts of interest

On behalf of all authors, the corresponding author states that there is no conflict of interest.

## Supplementary Material

RA-015-D5RA03467A-s001

## Data Availability

The data that support the findings of this study are available from the corresponding author upon reasonable request. Supplementary information provides additional supporting figures and tables. See DOI: https://doi.org/10.1039/d5ra03467a.
