# Supplementary material for: Green synthesis of iron-doped cobalt sulfide via synergistic electronic and structural engineering in ethaline deep eutectic solvent for efficient oxygen evolution reaction
Source: RSC Adv. 2025 Sep 1;15(38):31095–106. doi: 10.1039/d5ra03467a (PMC12400507; doi:10.1039/d5ra03467a)
Supplement: RA-015-D5RA03467A-s001 [file RA-015-D5RA03467A-s001.pdf]

Supplementary Information (SI) for RSC Advances.
This journal is © The Royal Society of Chemistry 2025

Supporting materials

Green Synthesis of Iron-Doped Cobalt Sulfide via Synergistic Electronic and Structural Engineering in Ethaline deep eutectic solvent for Efficient Oxygen Evolution Reaction

Wenqiang Yang, [a,b,1] Shaohua Wang,[a] Wen Shi,[a] Yakun Yin,[a] Youpo Mise,[a] Juan An,[a,b] Xuejiao Zhou,[a,b] Wentang Xia, [a,b,1]

a School of Metallurgy and Power Engineering, Chongqing University of Science and Technology, Chongqing 401331, P.R. China

b Chongqing Municipal Key Laboratory of Institutions of Higher Education for Value-added Treatment and Green Extraction from Complicated Resources, Chongqing 401331, P.R. China

[1] Corresponding author. Tel: +86-023-65023711; Fax: +86-023-65023711.
E-mail address: wenqiangyang@cqust.edu.cn (Wenqiang Yang), wentangx@163.com (Wentang Xia)

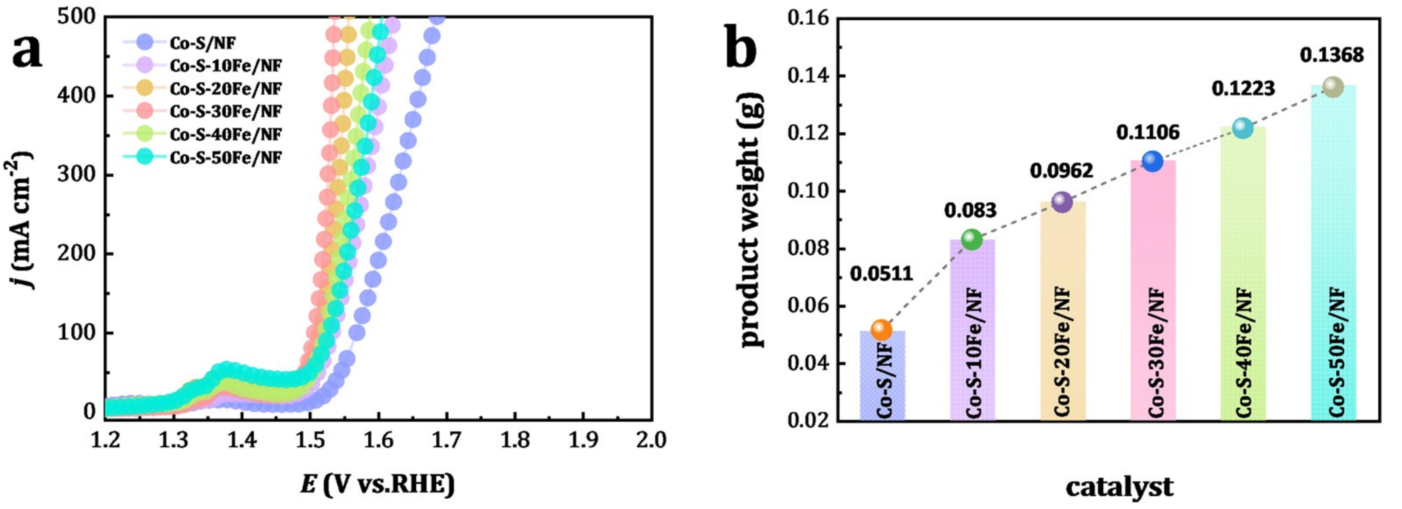

Figure S1. Polarization curves of Co-S (x=0) and Co-S-xFe (x=10, 20, 30, 40, 50) samples prepared from an Ethaline system containing 0.3 M $CoCl_2·6H_2O$, 0.2 M $Na_2S_2O_3$, and x mM $FeCl_3·6H_2O$, with a stirring rate of 300 rpm and a reaction time of 5 h. Measurements were performed in 1.0 M KOH at a scan rate of 5 mV s^{-1}, with 90% iR compensation.

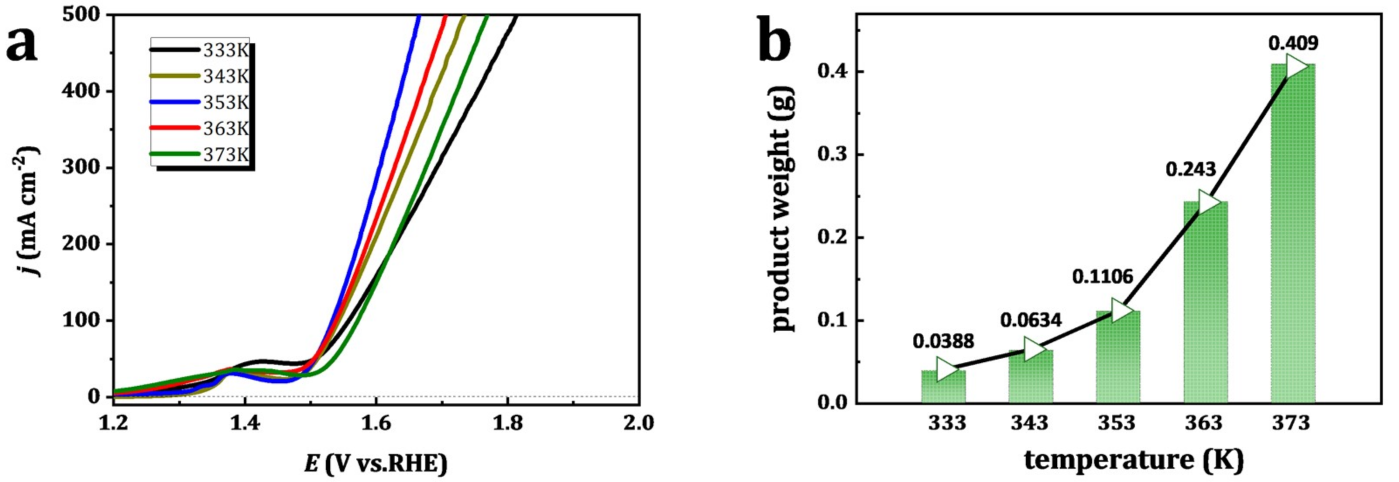

Figure S2. Polarization curves of Fe-doped Co-S samples obtained at various reaction temperatures (333, 343, 353, 363, and 373 K) from an Ethaline system containing 0.3 M $CoCl_2·6H_2O$, 0.2 M $Na_2S_2O_3$, and 50 mM $FeCl_3·6H_2O$, with a stirring rate of 300 rpm and a reaction time of 5 h. Measurements were conducted in 1.0 M KOH at a scan rate of 5 mV s^{-1} without iR compensation.

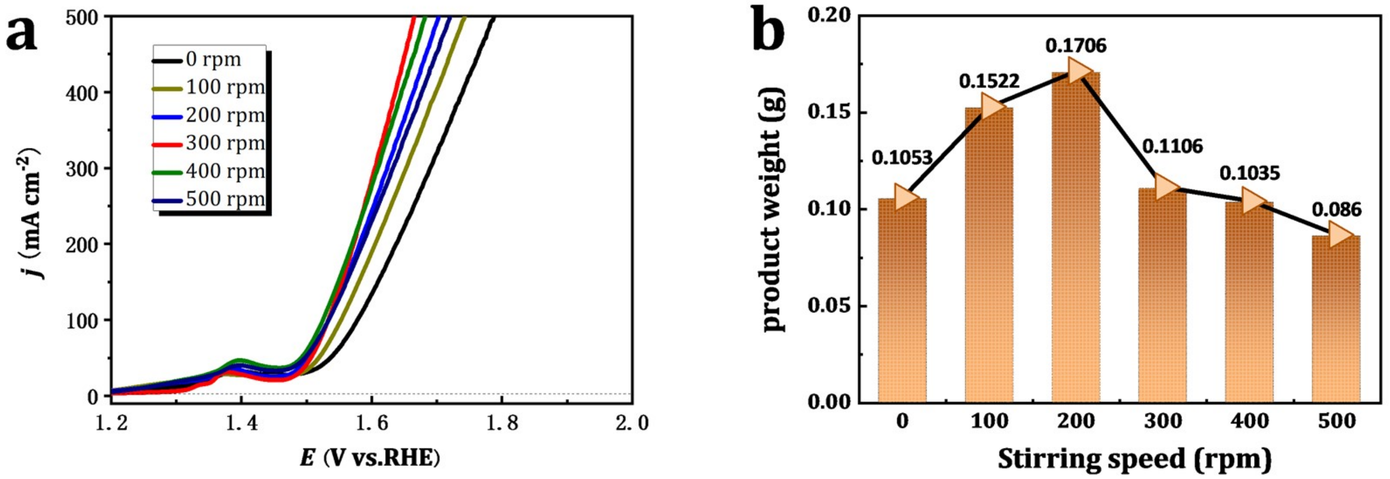

Figure S3. Polarization curves of Fe-doped Co-S samples obtained at various stirring rate (0, 100, 200, 300, 400, and 500 rpm) from an Ethaline system containing 0.3 M $CoCl_2·6H_2O$, 0.2 M $Na_2S_2O_3$ and 50 mM $FeCl_3·6H_2O$, with a reaction temperature of 353 K and a reaction time of 5 h. Measurements were conducted in 1.0 M KOH at a scan rate of 5 mV s^{-1} without iR compensation.

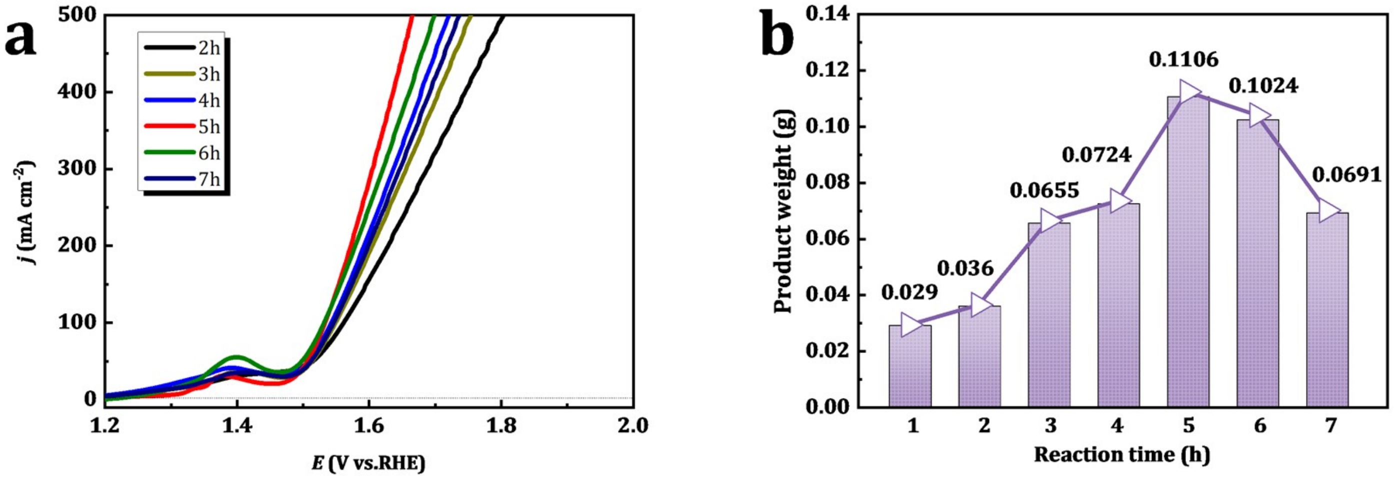

Figure S4. Polarization curves of the Fe-doped Co-S samples obtained at various reaction time (0, 1, 2, 3, 4, 5, 6, and 7 h) from an Ethaline system containing 0.3 M $CoCl_2·6H_2O$, 0.2 M $Na_2S_2O_3$ and 50 mM $FeCl_3·6H_2O$, with a reaction temperature of 353 K and a stirring rate of 300 rpm. Measurements were conducted in 1.0 M KOH at a scan rate of 5 mV s^{-1} without iR compensation.

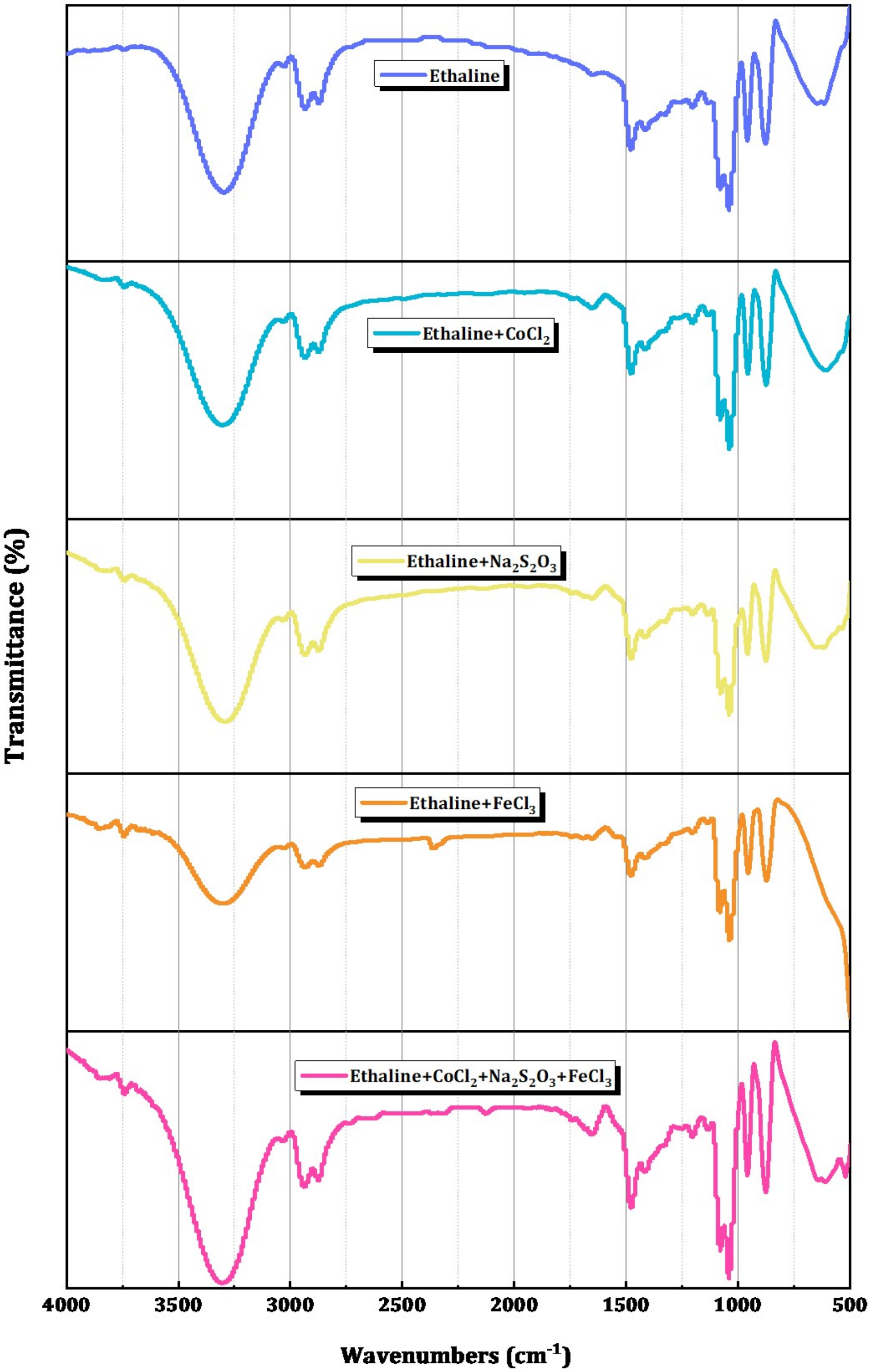

Fig. S5. FTIR spectra for pure Ethaline, single-solute systems of 0.30 M $CoCl_2$-Ethaline, 0.20 M $Na_2S_2O_3$-Ethaline, and 0.03 M $FeCl_3$-Ethaline, and a binary system of 0.30 M $CoCl_2$-0.20 M $Na_2S_2O_3$-0.03 M $FeCl_3$-Ethaline.

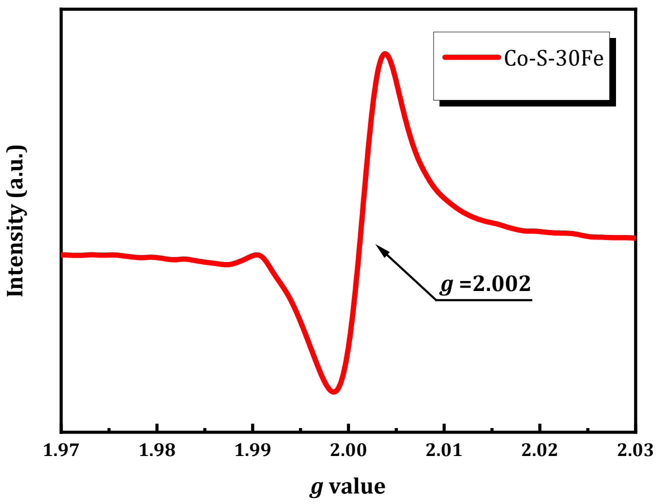

Fig. S6. low-temperature (77 K) EPR spectra of Co-S-30Fe sample.

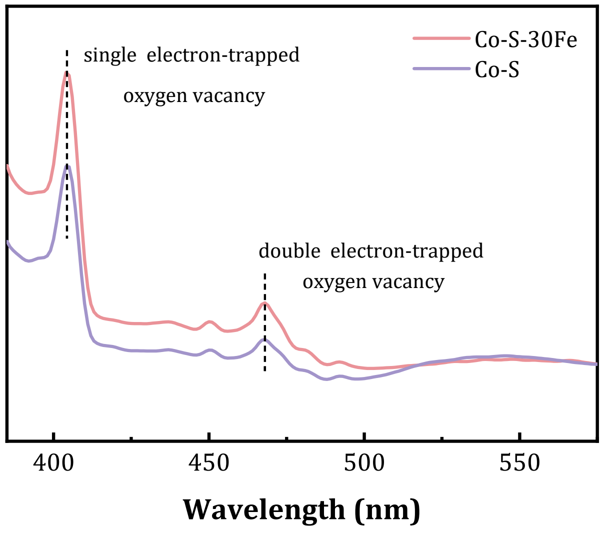

Fig. S7. Comparison of photoluminescence (PL) spectra of the Co-S and Co-S-30Fe.

As shown in Fig. S7, the PL spectrum exhibits two peaks. The strong peak at 404.3 nm corresponds to the recombination of photogenerated holes with single-electron-trapped oxygen vacancies ($V_O^{\cdot}$), while the peak at around 468.0 nm is associated with the recombination of holes with two-electron-trapped oxygen vacancies ($V_O^{\cdot\cdot}$) [1-4].

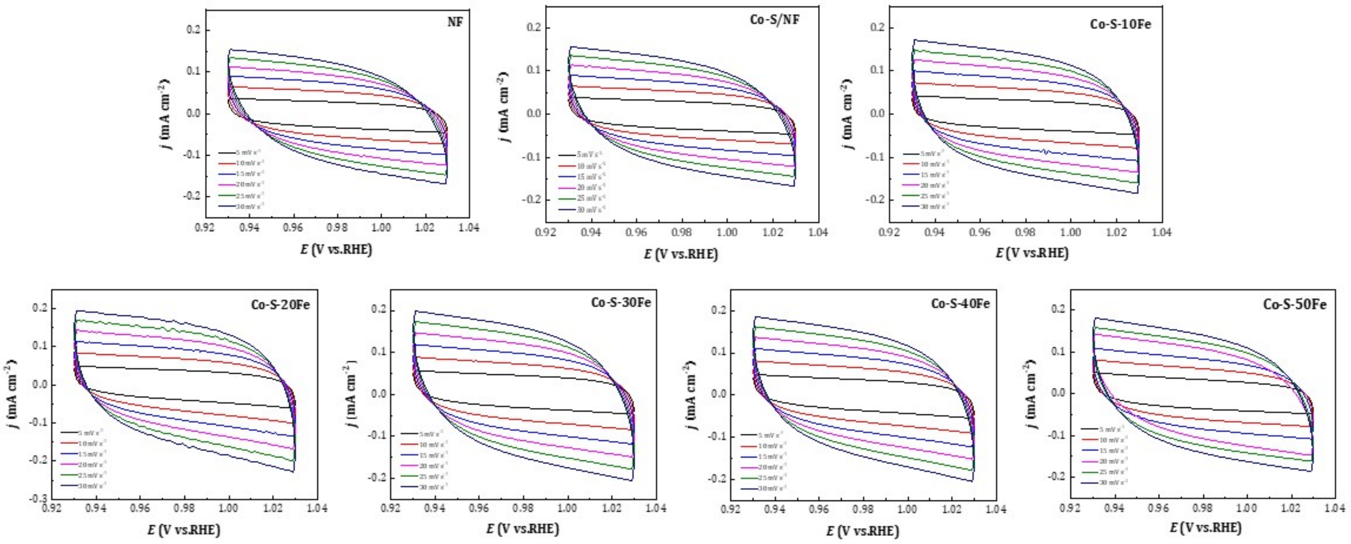

Figure S8. CVs obtained in the non-Faraday region of 0.93-1.03 V vs. RHE for Co-S/NF, Co-S-xFe/NF (x=10, 20, 30, 40, 50, as indicated) and bare NF.

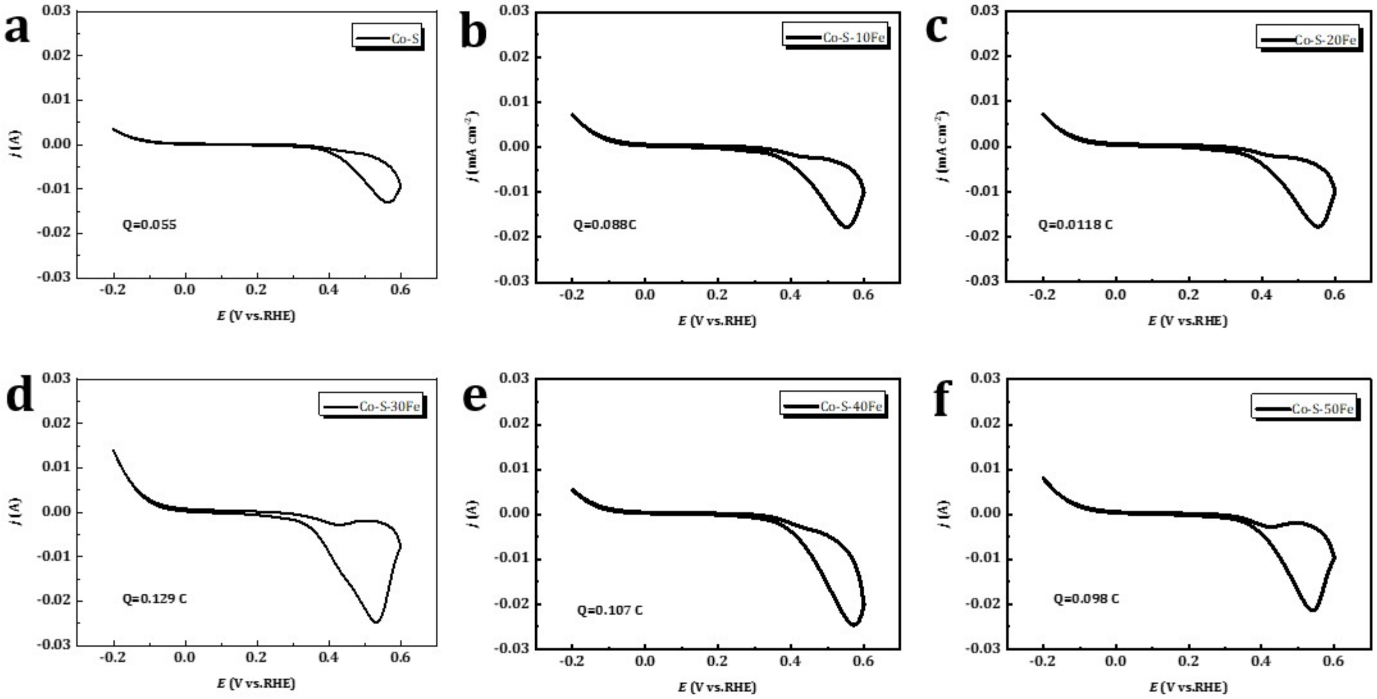

Figure S9. CVs of Co-S/NF, Co-S-xFe/NF (x=10, 20, 30, 40, 50, as indicated) between -0.2~0.6 V vs.RHE in 2.0 M PBS solution (pH=7.0) with a scan rate of 50 mV s^{-1}.

The TOF values of Co-S/NF and Co-S-xFe/NF were calculated using the established formula [5, 6] for OER catalysts: TOF = I/(4×F×n), where I (A) represents the steady-state current at a selected overpotential (η), F is the Faraday constant (96485 C mol^{-1}), and n (mol cm^{-2}) denotes the electrochemically active site density on the electrode surface. The factor '4' accounts for the four-electron transfer process per O_2 molecule generated in the OER. The active site density (n) was experimentally determined by integrating the charge (Q, C) associated with cyclic voltammetry (CV) scans performed in 2.0 M PBS (pH 7.0), following the equation $n = Q/(4F)$ (as detailed in Section 2.3 and Fig. S9). This method is widely accepted for quantifying electrochemically accessible metal sites in cobalt-based OER catalysts and ensures that n reflects operando conditions rather than theoretical estimates.

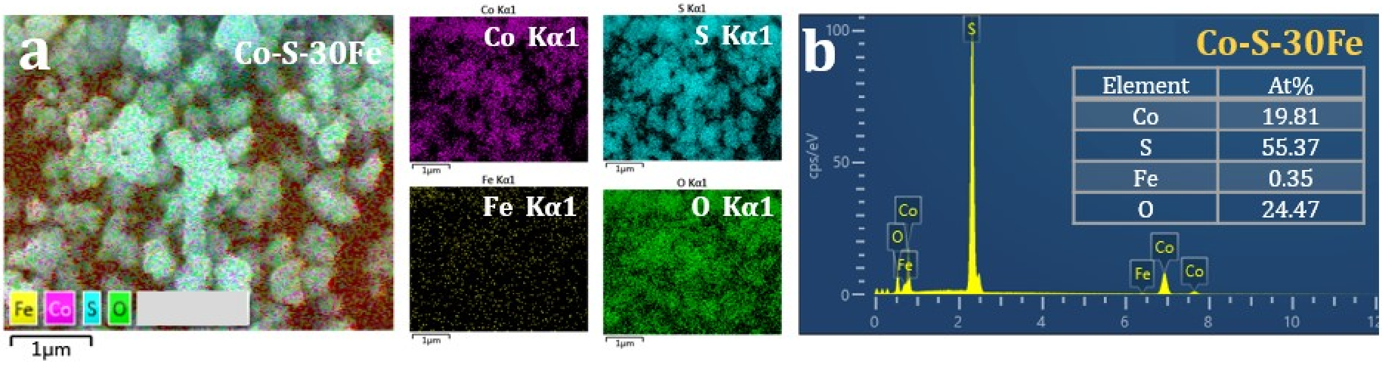

Fig. S10. (a) SEM image and elemental mapping images, and (b) EDS spectrum with the inset showing the atomic ratio for the Co-S-30Fe sample synthesized in Ethaline containing 0.20 M $CoCl_2$ and 0.05 mM $Na_2S_2O_3$ at 353 K for 6 h.

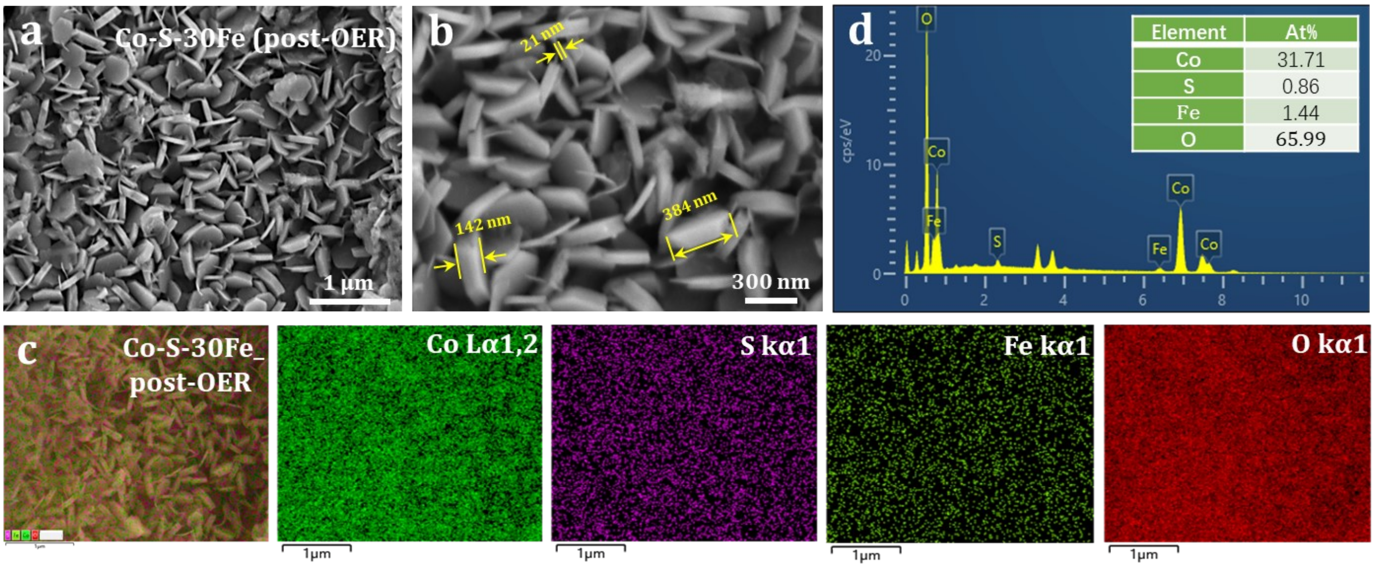

Fig. S11. (a-b) FESEM images of the post-OER Co-S-30Fe/NF at different magnifications. (c) Corresponding EDS elemental mapping images of Co, S, Fe, and O and (d) EDS spectrum with the inset table showing the molar contents for the post-OER Co-S-30Fe/NF sample.

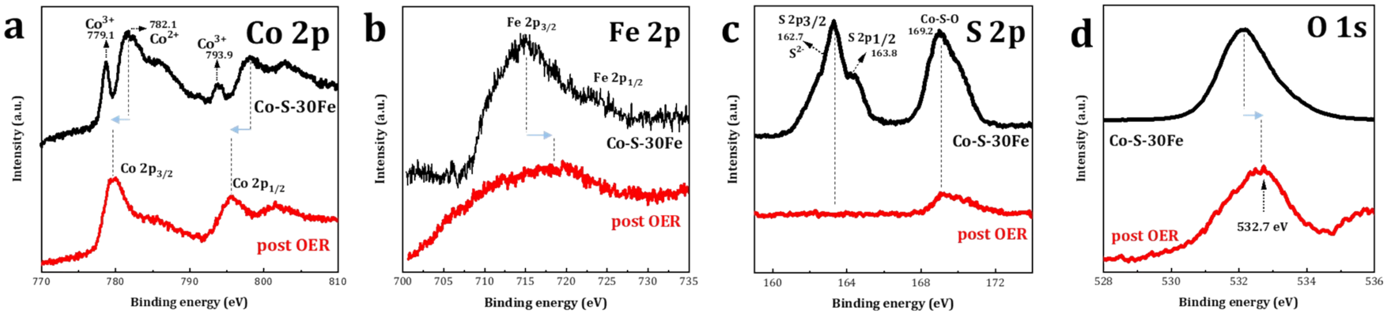

Fig. S12. Comparison of high-resolution XPS spectra of the pristine and post-OER Co-S-30Fe/NF samples (as indicated). (a) Co 2p, (b) Fe 2p, (c), and (d) O 1s spectra.

To gain deeper insights into the chemical state evolution of electrocatalysts during the OER, XPS analysis (Fig. S12) was performed on both pristine Co-S-30Fe/NF electrodes and those subjected to OER at 10 mA cm^{-2} in 1 M KOH for 27 h. The Co 2p spectra (Fig. S12a) reveal distinct negative shifts in the binding energies of the Co $2p_3/_2$ and Co $2p_1/_2$ peaks after OER, indicating an increased proportion of Co^{3+} species [7, 8]. Conversely, the Fe 2p spectra (Fig. S12b) exhibit positive shifts in the Fe $2p_3/_2$ and Fe $2p_1/_2$ peaks, signifying a higher concentration of Fe^{3+} [9, 10]. These opposing binding energy shifts collectively demonstrate the oxidation of both Co and Fe cations during OER, attributed to strong electronic interactions between Co(Fe)-S motifs and oxygen under highly oxidizing conditions. More pronounced changes are observed in the S 2p spectra (Fig. S12c), where the characteristic Co(Fe)-S peaks ($2p_3/_2$ at 162.7 eV and $2p_1/_2$ at 163.8 eV) completely disappear post-OER, accompanied by a significant reduction in the intensity of the S-O bond signal at 169.2 eV [11, 12]. These observations provide unambiguous evidence for sulfur dissolution and leaching from the electrode surface during OER. Furthermore, the O 1s spectra (Fig. S12d) show a marked positive shift (from 532.3 eV to 532.7 eV) after the reaction. Collectively, these XPS results demonstrate substantial electrochemical surface oxidation during OER. Oxygen incorporation displaces lattice sulfur atoms, leading to decomposition of Co(Fe)-S phases while promoting the formation of catalytically active (oxy)hydroxide species (Co(Fe)–O/OH) [12, 13]. This surface reconstruction process is consistent with the morphological and compositional transformation behavior observed in Fig. S11 and aligns with previously reported transformations in cobalt-based sulfide OER catalysts [12-14].

Table S1. ICP-OES surveys of the Co-S and Co-S-xFe (x=10, 20, 30, 40 and 50) samples.

Catalysts	S : Co (molar ratio)	S content (at.%)	Fe content (at.%)
Co-S	0.8	38.74	/
Co-S-10Fe	1.5	51.11	0.26
Co-S-20Fe	1.7	52.62	0.39
Co-S-30Fe	2.1	56.57	0.47
Co-S-40Fe	2.3	58.32	0.56
Co-S-50Fe	2.5	61.20	0.91

Table S2. Comparison of OER catalysts synthesized in DES and recently reported in 1.0 M KOH at 298 K.

Catalysts	Synthesis Method	Morphology	Overpotential (@10 mA cm^{-2}, mV)	Tafel slope/ (mV dec^{-1})	Ref.
Co-S-30Fe NPs	DES-assisted liquid-phase synthesis at 80 °C	nanospheres	230 η_{100}=278	44.6	This work
$NiFe_{0.05}$-N	DES solvothermolysis at 200 °C	2D nanosheets	238	76	[15]
$CoNi_2S_4$/CC	DES solvothermolysis at 180 °C	nanosheets	153 η_{100}=372	67.1	[16]
S-Mn/CP_Ac·H	Electrodeposition in DES at 80 °C	nano-flower	460	111.07	[17]
NiCo-500-15	Calcination at 500 °C	octahedra	320	67	[18]
$LaCoO_3$	Calcination at 900 °C	sub-micron particles	390	55.8	[19]
FeCoNiCrMo	Arc melting and DES corrosion	3D porous structure	280	105	[20]
Ni-Fe NPs/Fe	DES-induced galvanic replacement reaction at 80 °C	nanospheres	319	41.2	[21]
(FeCoNiCuZn)(C_2O_4)·$2H_2O$	DES solvothermolysis at 160 °C	nanosheets	265 η_{100}=334	45	[22]
Ni/$Ni(OH)_2$-Thr	DES solvothermolysis at 350 °C	nanospheres	270	40	[23]
S-MnO_x/Mn/CP	Electrodeposition in DES at 80 °C	nanoflakes	390	67	[24]
Ni/CP	Electrodeposition in DES at 60 °C followed by annealing at 400 °C	nanoparticles	380	60	[25]
NiFe@SS	Electrodeposition in DES at 85 °C	porous microspheres	234	45	[26]
Fe doped-$Ni(OH)_2$	Electrodeposition in DES at 25°C	nanoparticles	η_{100}=370	44	[27]
$Ni(OH)_2$/Ni/CF	Electrodeposition in DES at 60 °C	nanosheets	η_{100}=564	50	[28]
CoS_2/MS_2-HPMS	DES solvothermolysis at 130 °C followed by high-temperature sulfidation at 180 °C	hollow porous microspheres	217	65	[29]
PEG/TU-NiFeCoMnAl	DES solvothermolysis at 120 °C	nanosheets	220	39.7	[30]
NiCu@SS	Electrodeposition in DES at 30 °C	dendritic structure	η_{100}=447	75	[31]
SNO-C/Co_8FeS_8	Calcination at 900 °C	irregular particles	230	39.52	[32]
Ni-Fe/NF	Electrodeposition in DES at 30 °C	bulk alloy	219	74.38	[33]
$(NiFe)S_2$-3	DES solvothermolysis at 200 °C	hollow nanoparticles	257	41	[34]
NCD@NiFe-LDH	DES solvothermolysis at 120 °C	nanosheets@3D flowers	η_{500}=363 (0.1 M KOH)	49.8	[35]
$LiNi_{0.33}Co_{0.33}Mn_{0.33}O_2$	DES solvothermolysis at 200 °C	nanosheets@ microrods	273	50.46	[36]

Table S3. Fitted values of the EIS data of OER (η=350 mV) process (calculated from Fig. 5d).

**Catalysts**	**Solution series resistances** R_s **(ohm)**	**Charge transfer resistance** R_{ct} **(ohm)**
Co-S/NF	1.15	12.29
Co-S-10Fe/NF	1.20	9.17
Co-S-20Fe/NF	1.24	4.97
Co-S-30Fe/NF	1.17	4.14
Co-S-40Fe/NF	1.20	6.32
Co-S-50Fe/NF	1.15	8.03

References:

[1] J. Bao, X. Zhang, B. Fan, J. Zhang, M. Zhou, W. Yang, X. Hu, H. Wang, B. Pan, Y. Xie, Ultrathin Spinel-Structured Nanosheets Rich in Oxygen Deficiencies for Enhanced Electrocatalytic Water Oxidation, Angewandte Chemie, 127 (2015) 7507-7512.

[2] L. Zhuang, L. Ge, Y. Yang, M. Li, Y. Jia, X. Yao, Z. Zhu, Ultrathin Iron-Cobalt Oxide Nanosheets with Abundant Oxygen Vacancies for the Oxygen Evolution Reaction, Advanced Materials, 29 (2017) 1606793.

[3] W. Chen, T. Wang, J. Xue, S. Li, Z. Wang, S. Sun, Cobalt–nickel layered double hydroxides modified on TiO2 nanotube arrays for highly efficient and stable PEC water splitting, Small, 13 (2017) 1602420.

[4] F. Lei, Y. Sun, K. Liu, S. Gao, L. Liang, B. Pan, Y. Xie, Oxygen vacancies confined in ultrathin indium oxide porous sheets for promoted visible-light water splitting, J. Am. Chem. Soc., 136 (2014) 6826-6829.

[5] X.-L. Wang, L.-Z. Dong, M. Qiao, Y.-J. Tang, J. Liu, Y. Li, S.-L. Li, J.-X. Su, Y.-Q. Lan, Exploring the Performance Improvement of the Oxygen Evolution Reaction in a Stable Bimetal–Organic Framework System, Angewandte Chemie International Edition, 57 (2018) 9660-9664.

[6] D. Wang, C. Han, Z. Xing, Q. Li, X. Yang, Pt-like catalytic behavior of MoNi decorated CoMoO3 cuboid arrays for the hydrogen evolution reaction, Journal of Materials Chemistry A, 6 (2018) 15558-15563.

[7] Q. Zhang, N.M. Bedford, J. Pan, X. Lu, R. Amal, A Fully Reversible Water Electrolyzer Cell Made Up from FeCoNi (Oxy)hydroxide Atomic Layers, Advanced Energy Materials, 9 (2019) 1901312.

[8] P.W. Menezes, C. Panda, C. Walter, M. Schwarze, M. Driess, A Cobalt-Based Amorphous Bifunctional Electrocatalysts for Water-Splitting Evolved from a Single-Source Lazulite Cobalt Phosphate, Advanced Functional Materials, 29 (2019) 1808632.

[9] S.H. Oh, O.H. Kwon, Y.C. Kang, J.-K. Kim, J.S. Cho, Highly integrated and interconnected CNT hybrid nanofibers decorated with α-iron oxide as freestanding anodes for flexible lithium polymer batteries, Journal of Materials Chemistry A, 7 (2019) 12480-12488.

[10] Y. Wu, Y. Meng, J. Hou, S. Cao, Z. Gao, Z. Wu, L. Sun, Orienting Active Crystal Planes of New Class Lacunaris Fe2PO5 Polyhedrons for Robust Water Oxidation in Alkaline and Neutral Media, Advanced Functional Materials, 28 (2018) 1801397.

[11] C. Xuan, W. Lei, J. Wang, T. Zhao, C. Lai, Y. Zhu, Y. Sun, D. Wang, Sea urchin-like Ni–Fe sulfide architectures as efficient electrocatalysts for the oxygen evolution reaction, Journal of Materials Chemistry A, 7 (2019) 12350-12357.

[12] W. Yang, J. Zeng, Y. Hua, C. Xu, S.S. Siwal, Q. Zhang, Defect engineering of cobalt microspheres by S doping and electrochemical oxidation as efficient bifunctional and durable electrocatalysts for water splitting at high current densities, Journal of Power Sources, 436 (2019) 226887.

[13] W. Yang, Q. Zhang, S.S. Siwal, Y. Hua, C. Xu, Dynamic structure evolution of free-standing S-doped porous Co-Fe microspheres with enhanced oxygen evolution electrocatalysis in alkaline media, Electrochim. Acta, 361 (2020) 137038.

[14] L. Zheng, Y. Zhong, J. Cao, M. Liu, Y. Liao, H. Xu, S. Chen, F. Xiong, Y. Qing, Y. Wu, Modulation of electronic synergy to enhance the intrinsic activity of Fe5Ni4S8 nanosheets in restricted space carbonized wood frameworks for efficient oxygen evolution reaction, Small, 20 (2024) 2308928.

[15] Y. Xu, Z. Cheng, J. Jiang, J. Du, Q. Xu, 2D amorphous bi-metallic NiFe nitrides for a high-efficiency oxygen evolution reaction, Chem. Commun. (Cambridge, U. K.), 57 (2021) 13170-13173.

[16] X. Su, X. Shao, Y. Wang, W. Fan, C. Song, D. Wang, CoNi2S4 Nanosheets on Carbon Cloth Using a Deep Eutectic Solvent Strategy as Bifunctional Catalysts for Water/Simulated Seawater Electrolysis, ACS Applied Nano Materials, 6 (2023) 23029-23036.

[17] M. Guo, B. Zhang, Q. Zhang, Deep eutectic solvent mediated synthesis of Mn-based hybrid electrocatalyst for oxygen evolution reaction: Insights into the effect of anion on the evolution of structure-activity, Appl. Surf. Sci., 645 (2024) 158843.

[18] C. Zhang, B. Xin, T. Chen, H. Ying, Z. Li, J. Hao, Deep eutectic solvent strategy enables an octahedral Ni–Co precursor for creating high-performance NiCo2O4 catalyst toward oxygen evolution reaction, Green Energy & Environment, 7 (2022) 1217-1227.

[19] S. Hong, A.M. Díez, A.N. Adeyemi, J.P. Sousa, L.M. Salonen, O.I. Lebedev, Y.V. Kolen'ko, J.V. Zaikina, Deep eutectic solvent

synthesis of perovskite electrocatalysts for water oxidation, ACS Appl. Mater. Interfaces, 14 (2022) 23277-23284.
[20] Y.-c. Xu, W.-j. Chen, J.-f. Zhou, C.-b. Hu, S.-w. He, H. Liu, Z.-s. Hua, Deep Eutectic Solvent-Assisted Corrosion Boosting Bulk FeCoNiCrMo High-Entropy Alloys as Highly Efficient Oxygen Evolution Reaction Catalyst, Langmuir, 40 (2024) 14291-14302.
[21] R. Xue, M. Guo, Z. Wei, Q. Zhang, Deep eutectic solvent-induced synthesis of Ni–Fe catalyst with excellent mass activity and stability for water oxidation, Green Energy & Environment, 8 (2023) 852-863.
[22] H. Yang, Z. Cheng, P. Wu, Y. Wei, J. Jiang, Q. Xu, Deep eutectic solvents regulation synthesis of multi-metal oxalate for electrocatalytic oxygen evolution reaction and supercapacitor applications, Electrochim. Acta, 427 (2022) 140879.
[23] Y. Wei, J. Jiang, J. Dong, Y. Xu, J. Fu, Q. Xu, Designable synthesis of reactive deep eutectic solvents (RDESs) in regulating Ni-based materials for an efficient oxygen evolution reaction, Green Chem., 24 (2022) 8014-8020.
[24] M. Guo, Z. Wei, Q. Zhang, Electrochemical construction of S-doped MnOx/Mn integrated film on carbon paper in a choline chloride based deep eutectic solvent for enhanced electrochemical water oxidation, Int. J. Hydrogen Energy, 47 (2022) 6029-6043.
[25] S. Elsharkawya, S. Hammad, I. El-hallaga, Electrodeposition of Ni nanoparticles from deep eutectic solvent and aqueous solution promoting high stability electrocatalyst for hydrogen and oxygen evolution reactions, Journal of Solid State Electrochemistry, 26 (2022) 1501-1517.
[26] P.M. Viyanni, T.N.J.I. Edison, M.G. Sethuraman, Electrosynthesis of deep eutectic solvent assisted NiFe nanoparticles on stainless steel mesh for electrocatalytic water splitting reactions, Materials Today Sustainability, 24 (2023) 100565.
[27] Q. Li, M. Gao, M. Cheng, H. Li, Y. Hua, Q. Zhang, J. Ru, Fine-controlled oxygen vacancies of nanoporous Fe doped-Ni (OH) 2 composite films enabled by H2O regulated deep eutectic solvent for alkaline oxygen evolution reaction at industrial current densities, Electrochim. Acta, 478 (2024) 143859.
[28] Y.H. Liu, H.-W. Guo, F.-Y. Zeng, Green fabrication of nanostructured Ni (OH) 2/Ni/Carbon felt electrodes with water-containing deep eutectic solvent for enhanced water electrolysis performance, J. Power Sources, 570 (2023) 233043.
[29] X. Wang, L. Wang, K.K. Rani, X. Peng, Y. Ning, X. Liu, Y. Fan, D.-H. Chen, W. Chen, Heterogeneous CoS 2/MS 2 microspheres for an efficient oxygen evolution reaction, Inorganic Chemistry Frontiers, 11 (2024) 98-106.
[30] S. Guan, B. Xu, J. Wu, J. Han, T. Guan, Y. Yang, K. Li, J. Wang, High-entropy materials based on deep eutectic solvent for boosting oxygen evolution reaction, Fuel, 358 (2024) 130315.
[31] P.M. Viyanni, M.G. Sethuraman, Influence of diverse hydrogen bond donors in deep eutectic solvents on the electrochemical deposition of NiCu alloy on stainless steel mesh: Exploring NiCu efficacy in water splitting and methanol oxidation reactions, Int. J. Hydrogen Energy, 130 (2025) 615-630.
[32] Y. Ye, X. Zhao, G. Wei, S. Gu, C. Li, H. Zhang, J. Zhang, X. Li, H. Chen, Multiscale regulation of S, N, O tri-doped carbon/Co 8 FeS 8 catalysts with SO 4 2−-riched and lattice distortion for efficient water splitting, J. Mater. Chem. A, 12 (2024) 27724-27731.
[33] Y. He, Z. Wang, Z. Hu, Y. Hu, X. Fan, S. Liu, C. Wang, Nickel-iron alloy films electrodeposited from metal salt-l-serine deep eutectic solvent for alkaline oxygen evolution reaction, Journal of Colloid and Interface Science, (2025) 137911.
[34] H. Zhao, W. Li, R. Wang, One-pot synthesis and microstructure analysis of Fe-doped NiS 2 for efficient oxygen evolution electrocatalysis, Materials Advances, 3 (2022) 7125-7131.
[35] Z. Hu, D. Zhang, C. Sun, C. Song, D. Wang, One-step ionothermal accompanied thermolysis strategy for N-doped carbon quantum dots hybridized NiFe LDH ultrathin nanosheets for electrocatalytic water oxidation, Electrochim. Acta, 391 (2021) 138932.
[36] M. Chen, Y. Zhou, L. Wang, G. Xue, J. Guo, Y. Wang, Recycling spent LiNi 1− x− y Co x Mn y O 2 cathodes to efficient catalysts for the oxygen evolution reaction, Green Chem., 26 (2024) 2912-2921.
